# Supplementary material for: The conserved N-terminal histidine in an engineered peptide mediates sepsis treatment efficacy via dual binding to CD14 and LPS
Source: Mol Ther. 2025 Sep 23;34(1):407–22. doi: 10.1016/j.ymthe.2025.09.033 (PMC12925814; doi:10.1016/j.ymthe.2025.09.033)
Supplement: Document S1. Figures S1–S15 [file mmc1.pdf]

YMTHE, Volume *34*

Supplemental Information

The conserved N-terminal histidine in an engineered peptide mediates sepsis treatment efficacy via dual binding to CD14 and LPS

Ganna Petruk, Firdaus Samsudin, Manoj Puthia, Jitka Petrlova, Peter J. Bond, and Artur Schmidtchen

Supplemental Figures

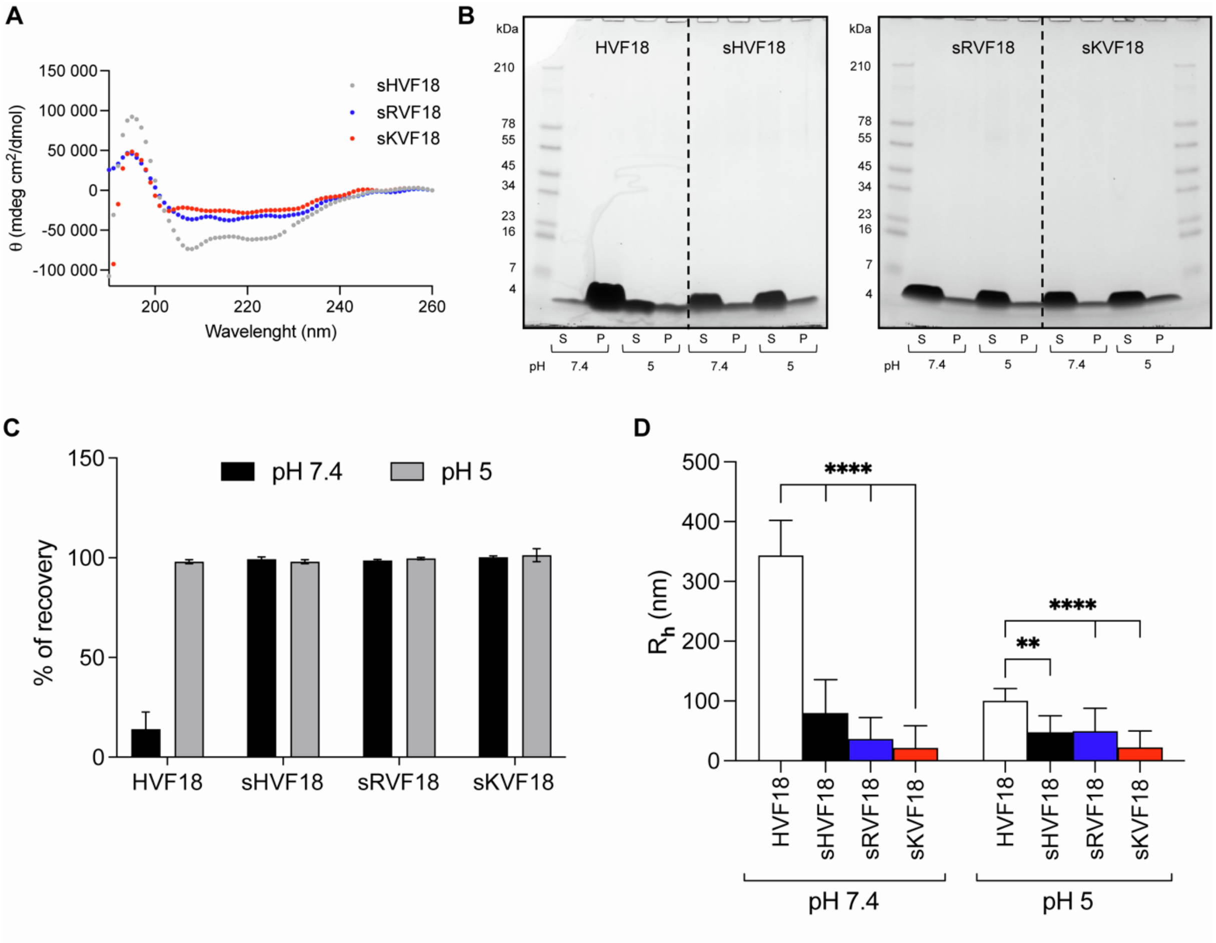

Figure S1 | Solubility of sHVF18 and its variants. (A) Circular dichroism spectra of sHVF18, sRVF18 and sKVF18 dissolved in 10 mM NaOAc at pH 5.0. Data are shown as mean ± SD of 3 different experiments (n = 3). (B) SDS-PAGE of supernatants (S) and pellets (P) from the centrifugation of 30 µL (1 mM) sHVF18 and its variants dissolved in 10 mM Tris at pH 7.4 or 10 mM NaOAc pH 5.0. (C) The graph shows the percentage of peptide recovery ± SD in the supernatant after centrifugation with respect to the starting concentration. (D) Peptides were dissolved in 10 mM Tris at pH 7.4 or 10 mM NaOAc pH 5.0 at 1 mM as final concentration, then hydrodynamic radii of the particles in solution were measured by DLS. The data are presented as mean ± SD (n = 3).

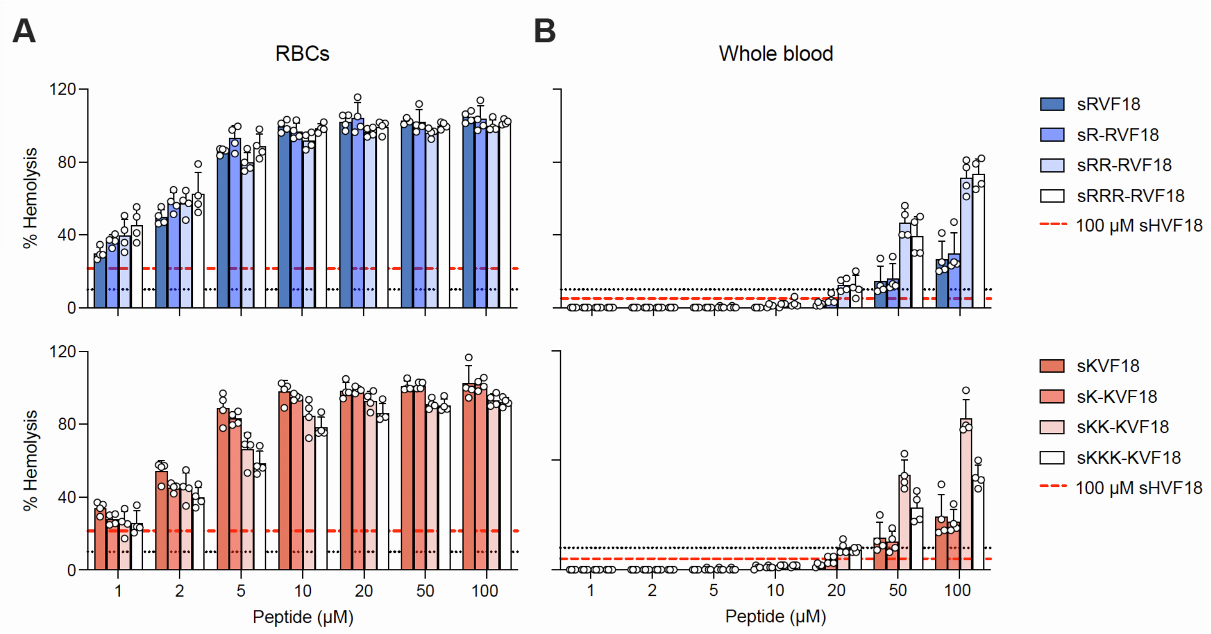

Figure S2 | Hemolytic effect of different sHVF18 variants. (A) The curves show the hemolytic activity of the peptides on erythrocytes (RBCs, A) or whole blood (B). Results are presented as means ± SD of four different experiments (n = 4). For each experiment, blood from a different donor was used. Red dashed line shows the hemolysis induced by 100 μM sHVF18. Black dotted line shows 10% of hemolysis.

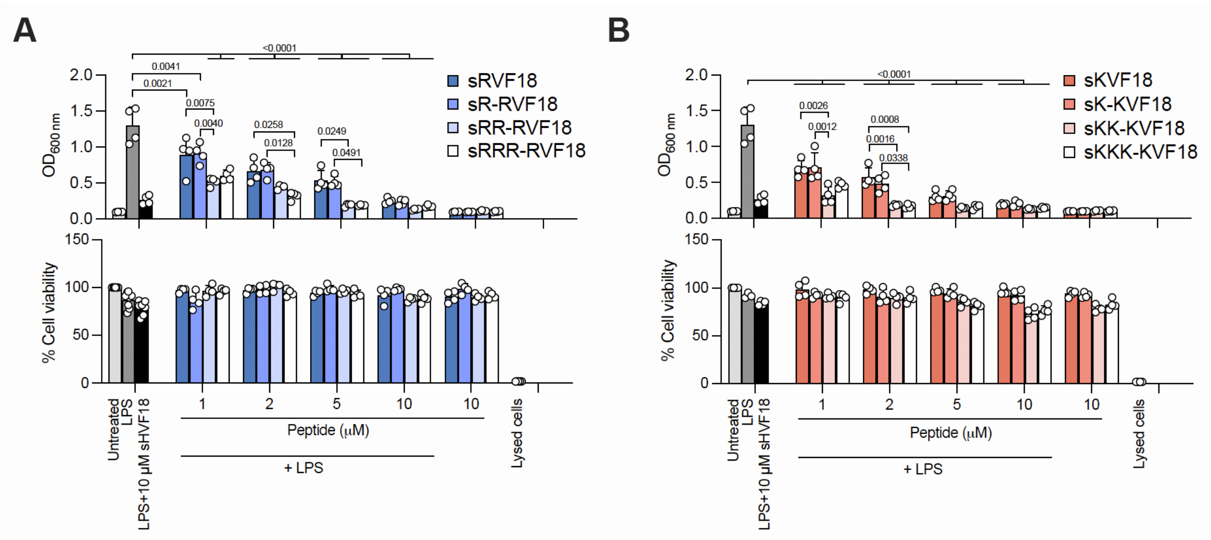

Figure S3 | Anti-inflammatory activity of different sHVF18 variants in THP1-XBlue-CD14 reporter cells. NF-κB activation and cell viability using monocytes stimulated with 100 ng mL^{-1} *E. coli* LPS in the presence or absence of increasing concentrations of arginine (A) or lysin (B) variants of sHVF18, 20 h poststimulation. Results are presented as means ± SD of four experiments (n = 4). P values were determined using an ordinary two-way ANOVA followed by Tukey's multiple comparisons tests using GraphPad Prism software.

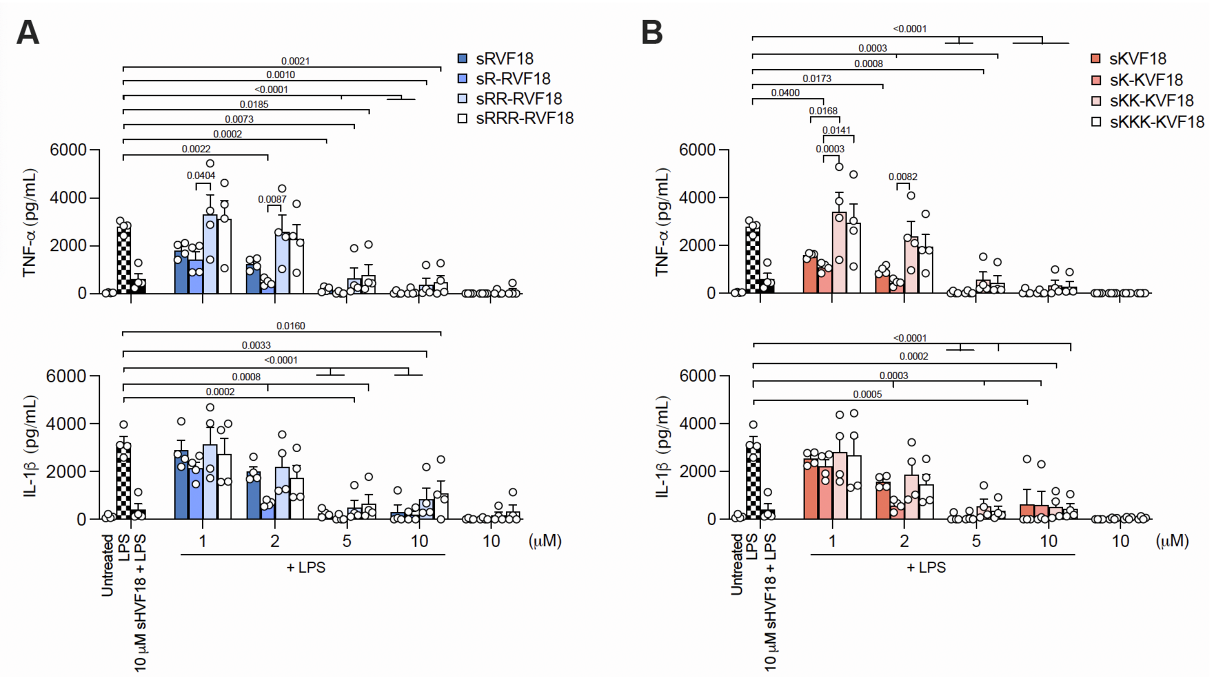

Figure S4 | Anti-inflammatory activity of different sHVF18 variants in human blood. TNF-α and IL-1β release from blood stimulated with 100 ng mL^{-1} *E. coli* LPS in the presence or absence of increasing concentrations of arginine (A) or lysin (B) variants of sHVF18, 24 h poststimulation. Results are presented as means ± SEM of four experiments, each performed with the blood from a different donor (n = 4). P values were determined using an ordinary two-way ANOVA followed by Tukey's multiple comparisons tests using GraphPad Prism software.

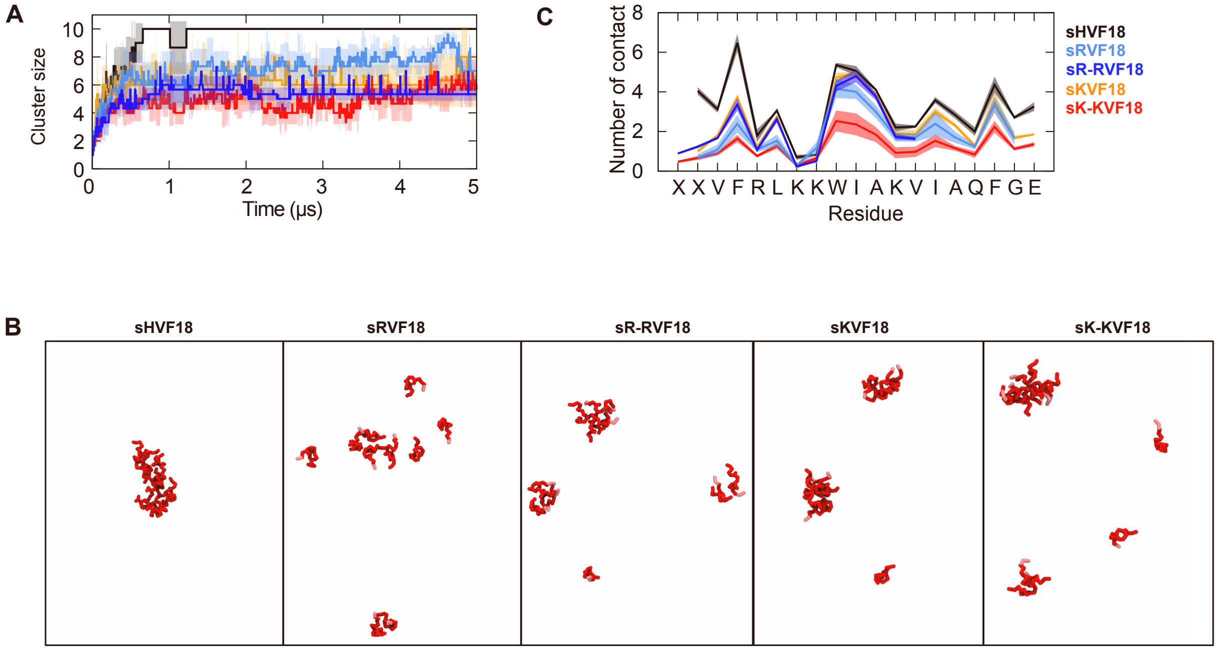

Figure S5 | sHVF18 variants with positively charged N-terminus aggregate less in solution. (A) CG simulations of 10 copies of sHVF18 or N-terminal variant peptides in a box of water were performed. The graph shows comparison of the number of peptides forming the largest cluster throughout the simulations, whereby a cluster size of 10 indicates that all peptides form one cluster. Average values from three independent repeats are shown as thick lines, whereas the standard deviations are depicted as shaded areas. Cut-off distance for cluster size calculation is 0.6 nm. (B) The final snapshots from one of the simulations for each peptide. Peptides are coloured in red with the N-terminal arginine and lysine highlighted in pink. (C) Average number of contacts made by each residue of one peptide with all the other peptides in the system in the last 3 μs of the simulations. Cut-off distance for contact calculation is 0.6 nm.

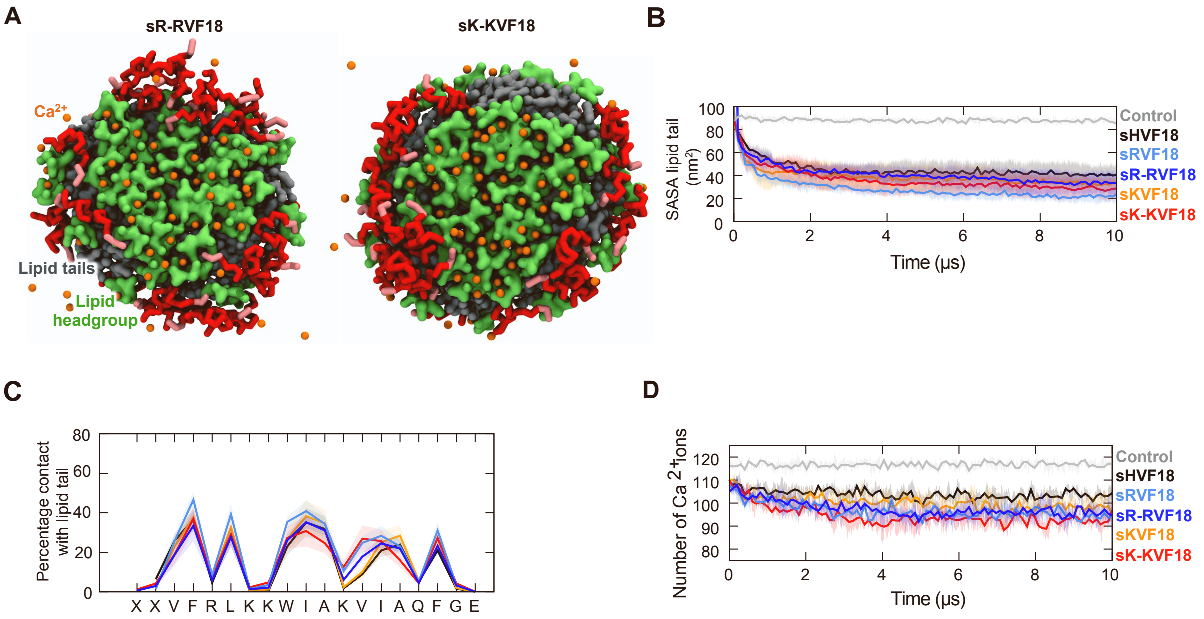

Figure S6 | CG simulations of sHVF18 and N-terminal variants with lipid A aggregate. (A) The final snapshots from one CG simulations of lipid A aggregate with sR-RVF18 and sK-KVF18. Peptides are coloured in red with the N-terminal arginine or lysine residues highlighted in pink. The lipids are shown in surface representation with the headgroup in green and tails in grey. Ca^{2+} ions are shown as orange spheres. (B) Solvent accessible surface area (SASA) of lipid tails throughout the simulations with peptides and without peptides (grey). Thick lines show average over three independent simulations and the standard deviation is depicted as the shaded areas. A probe radius of 0.26 nm was used for the calculation to represent the radius of water particles in the Martini force field. (C) Percentage of contact made by each residue of the peptide with lipid tails. Cut off distance for contact measurement is 0.6 nm. (D) The number of Ca^{2+} ions found within 0.6 nm of the phosphate groups of the lipids.

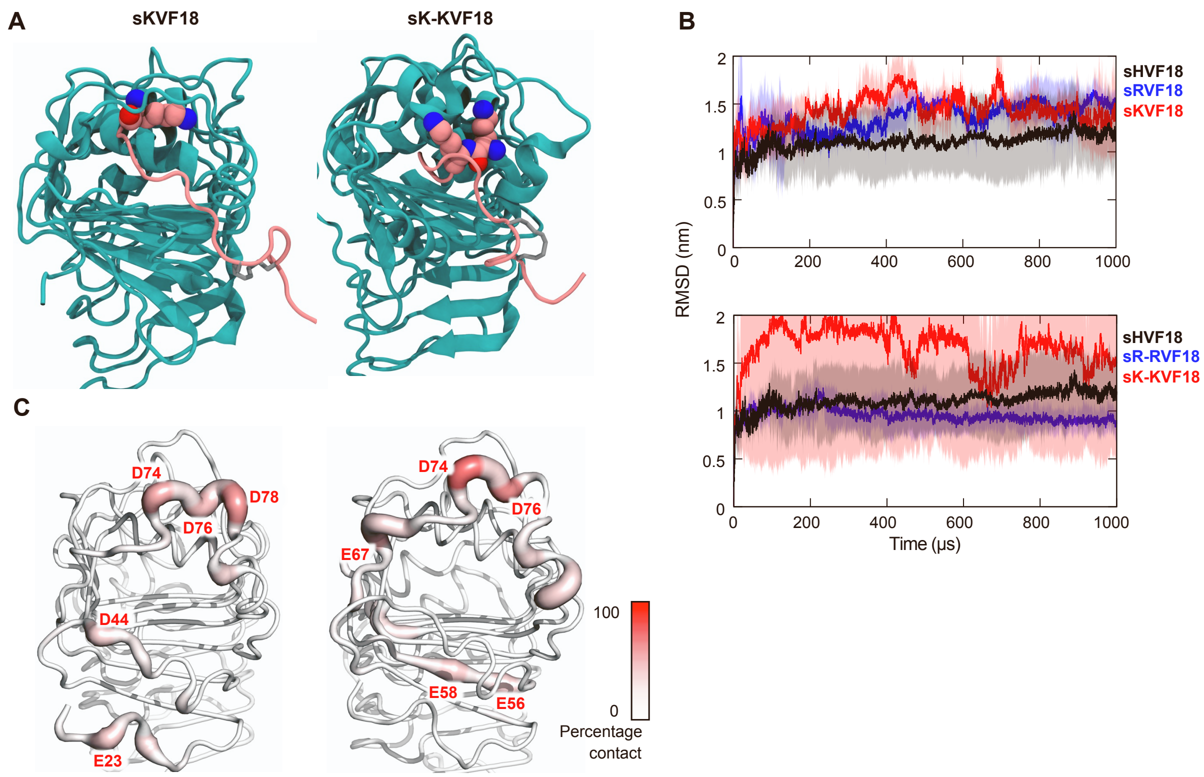

Figure S7 | Simulations of modified peptides bound to CD14. (A) The N-terminal histidine residue was mutated to either one or two lysine residues and three independent 1 µs simulations were performed. The figures show CD14 (cyan) and peptide (pink) with the N-terminal lysine residue(s) depicted in van der Waals representation, taken from the central structure of the top cluster from a clustering analysis performed on concatenated trajectories. (B) RMSD of backbone atoms of the whole peptide after least square fitting to the initial structure of the CD14-peptide complex, compared to the RMSD of sHVF18 (black). Thick lines show average values from three simulations, and the standard deviations are shown as shaded regions. (C) Average percentage of contacts made by CD14 residues with the ε-ammonium group of lysine at the N-terminus of modified peptides from three simulations. The top five residues that made the most contacts are labelled.

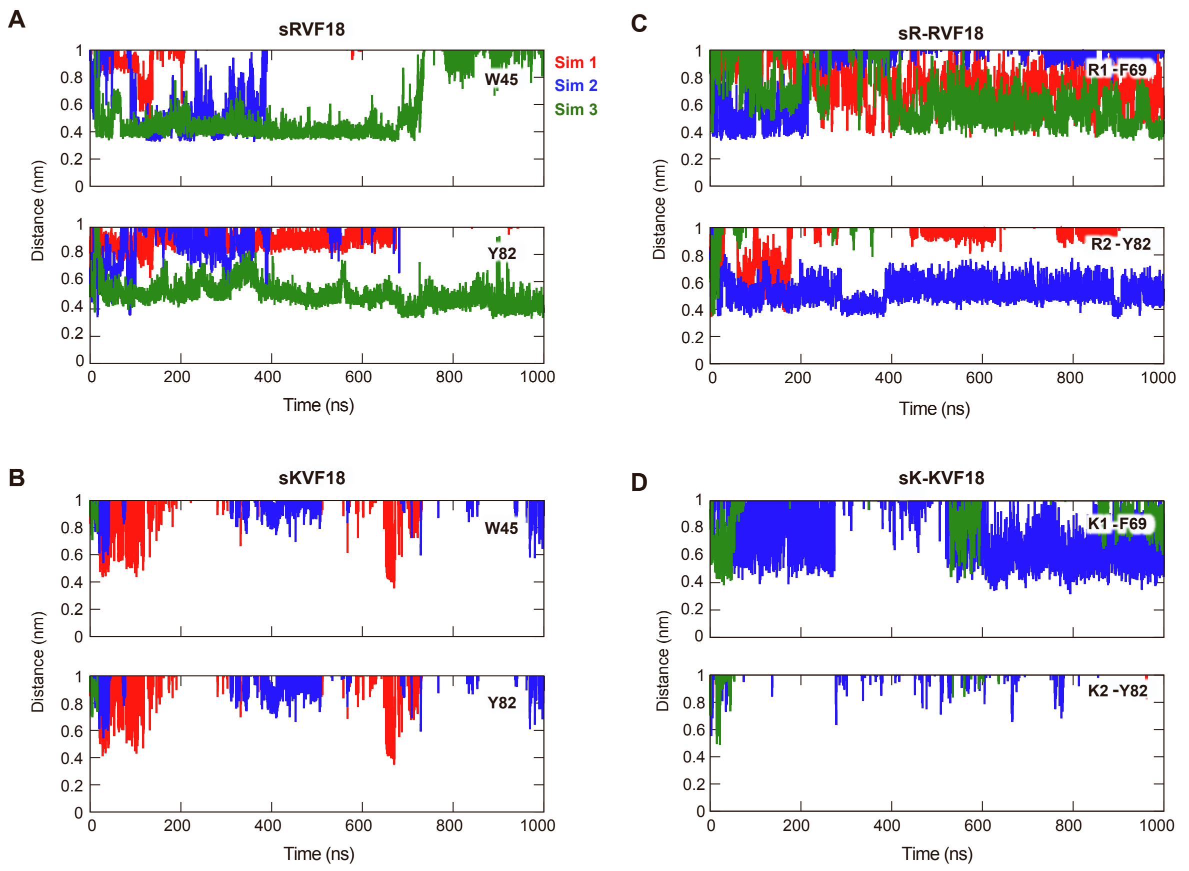

Figure S8 | Distance measurement between N-terminal arginine and lysine residues and aromatic residues on CD14. (A) The distance from the guanidinium group of residue R1 on sRVF18 with the side chain of residues W45 and Y82 on CD14 from three independent simulations. (B) Similar distance measurement performed for the ε-ammonium group of K1 residue on sKVF18. (C) The distance from the guanidinium group of residue R1 (top) and R2 (bottom) on sR-RVF18 with the side chain of residues F69 and Y82, respectively, on CD14 from three independent simulations. (D) Similar distance measurement performed for the ε-ammonium group of N-terminal lysine residues on sK-KVF18.

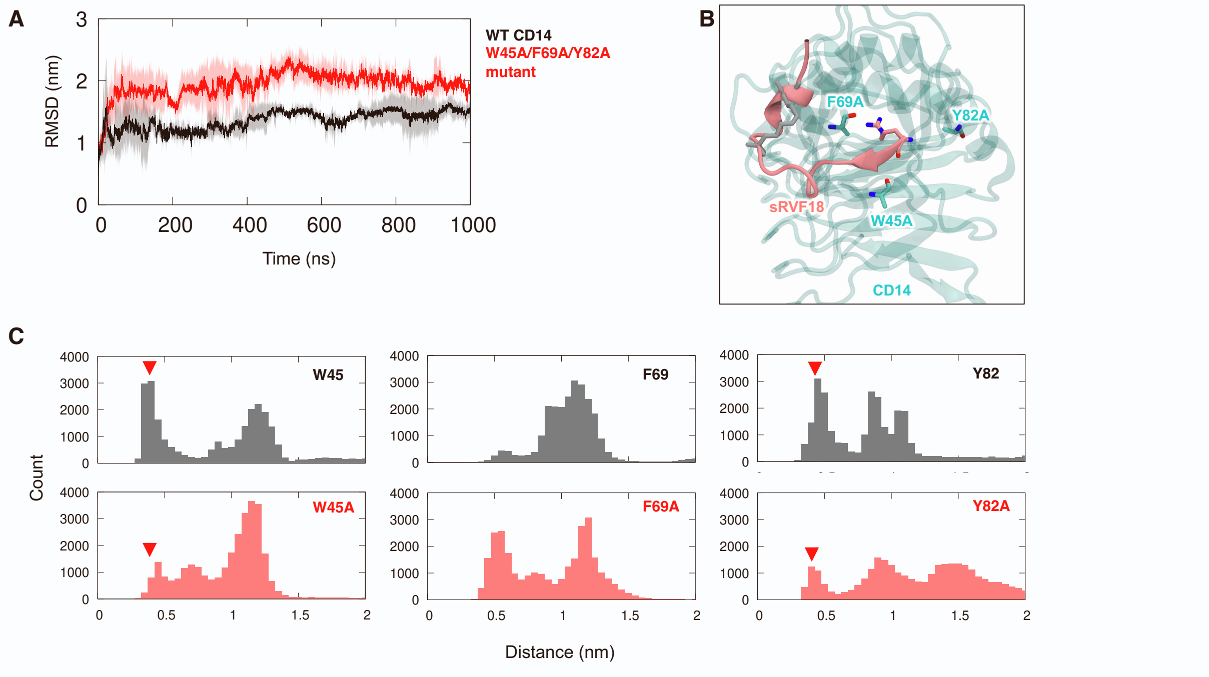

Figure S9 | Simulation of sRVF18 with CD14 mutant. (A) Residues W45, F69, and Y82 on CD14 were mutated to alanine and all-atom MD simulations of CD14 mutant complexed with sRVF18 were performed. The figure shows average RMSDs of backbone atoms of the peptide, comparing simulations with wild-type CD14 (black) and the mutant (red). The RMSDs are calculated after least square fitting to the initial structure of the CD14-peptide complex. The values are averaged from three independent simulations, while the standard deviation is shown as shaded areas. (B) Cluster analysis was performed on concatenated trajectories using an RMSD cut-off of 0.4 nm. The figure shows the central structure of the top cluster with CD14 in cyan and the peptide in pink. The N-terminal arginine as well as mutated residues on CD14 are highlighted in stick representation. (C) The distribution of distance between the side chain of N-terminal arginine on sRVF18 and the side chain of W45, F69, and Y82 from wild-type simulations (black) compared to the alanine mutant (red). Red triangles highlight the loss of interaction between the arginine side chain and residues W45 and Y82 in mutant simulations. The distance values are cumulative from three independent simulations.

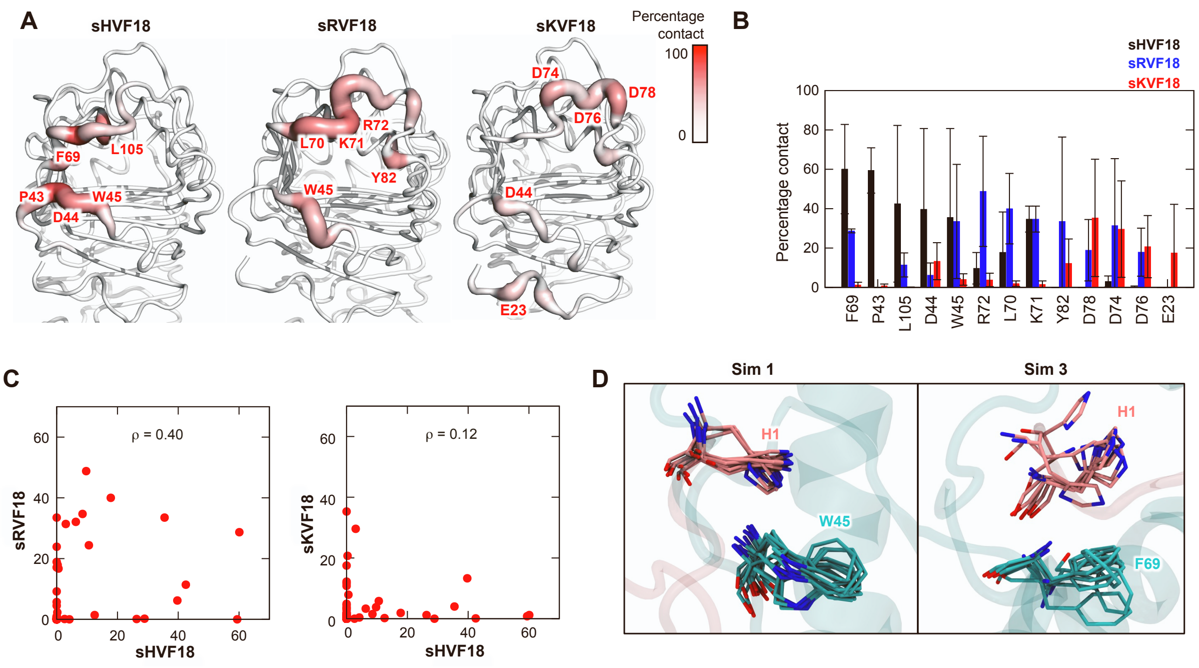

Figure S10 | Comparison of contacts made by sHVF18, sRVF18 and sKVF18 peptides on CD14. (A) Average percentage of contacts made by each residue on CD14 to the imidazole group of histidine, the guanidinium group of arginine, and the ε-ammonium group of lysine at the N-terminus of the respective peptides from three independent 1 μs simulations. The top five residues that made the most contacts are labelled. (B) The values of percentage of contacts made with the top five residues. Error bars show standard deviation between three repeats. (C) Correlation plot of average percentage contacts made by N-terminal residues of sHVF18 with either sRVF18 (left) or sKVF18 (right). The Pearson correlation coefficient values are shown. (D) Overlaid snapshots from sHVF18-CD14 simulations taken every 50 ns from parts of the simulations showing staggered π-π stacking interaction between the N-terminal histidine residue on the peptide (pink) and aromatic residues on CD14 (cyan).

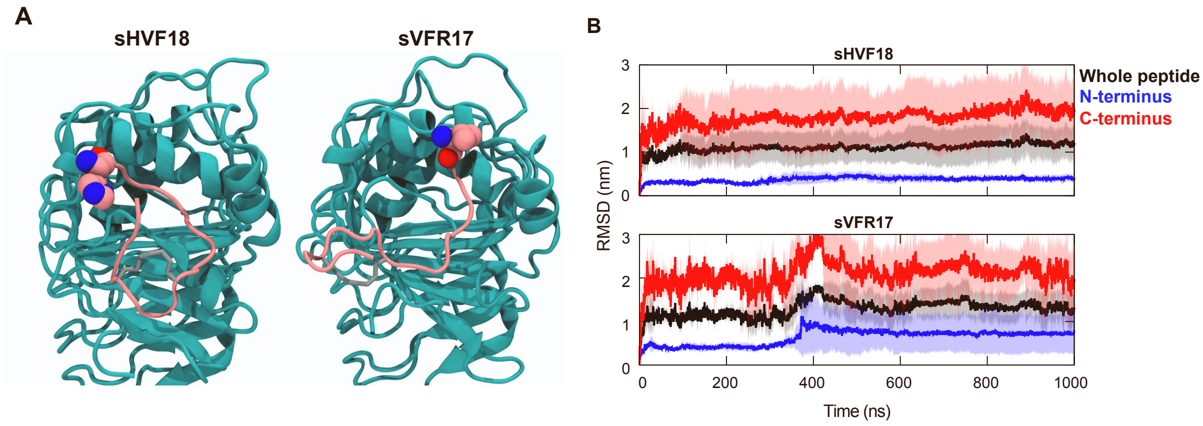

Figure S11 | All-atom MD simulations of CD14 bound to sHVF18 and sVFR17. (A) The figure shows a snapshot of CD14 (cyan) and peptide (pink) with the N-terminal H1 or V1 residue shown in van der Waals representation. The snapshot is taken from the central structure of the top cluster from a clustering analysis performed on concatenated trajectories. (B) RMSD profiles of the modelled sVFR17 compared to sHVF18. The figure shows the RMSD of backbone atoms of the first five residues at the N-terminus (blue), the last five residues at the C-terminus (red), and all residues (black) after least-squares fitting to the backbone atoms of the CD14-peptide complex. Thick lines show average values from three simulations, and the standard deviations are shown as shaded regions.

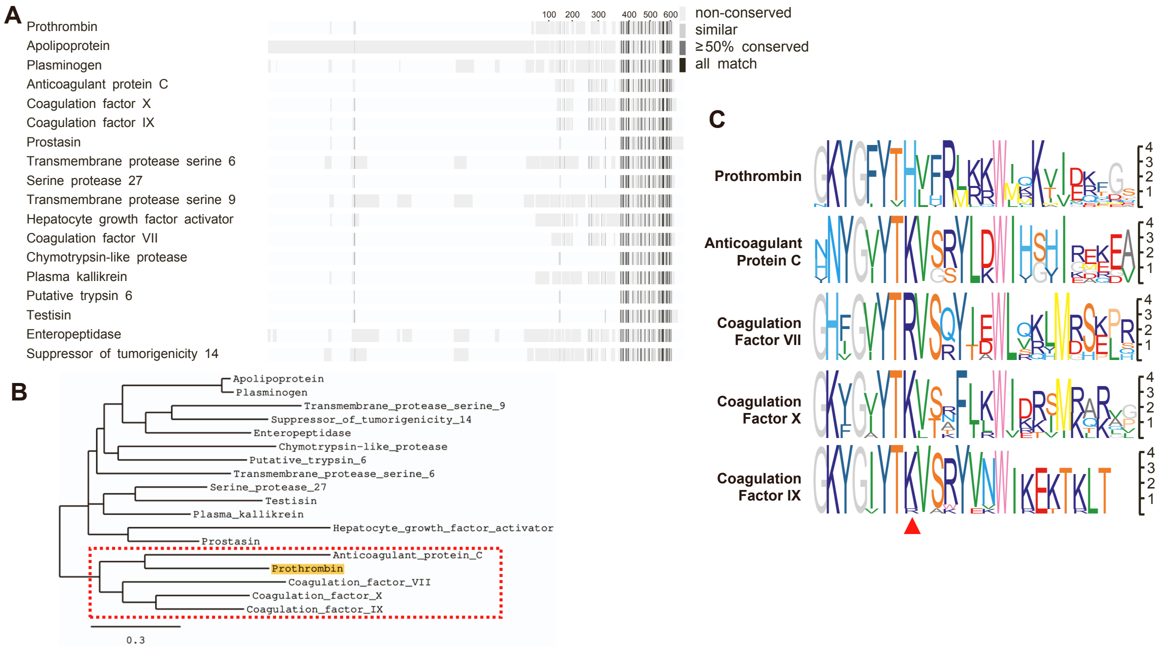

Figure S12 | Histidine is not conserved in thrombin homologs. (A) The sequence of human prothrombin (UniProt ID: P00734) was used to search for similar proteins in human in the UniProt database using the protein-protein BLAST algorithm (blastp). The figure shows sequence alignment of these proteins as fingerprints, whereby conserved regions are shown in dark grey. (B) Phylogenetic tree of prothrombin and related proteins based on the sequence alignment built using PhyML and TreeDyn algorithms (www.phylogeny.fr). Proteins that are in the same tree branch as prothrombin are highlighted in the dotted red box. (C) Sequences from different organisms for each protein in the same tree branch as prothrombin were searched on the UniProt database using the blastp algorithm and aligned to the sequences of prothrombin. The figure shows consensus sequence in WebLogo representation for residues that aligned with TCP25. The position of N-terminal histidine residue in HVF18 peptide is indicated by the red triangle.

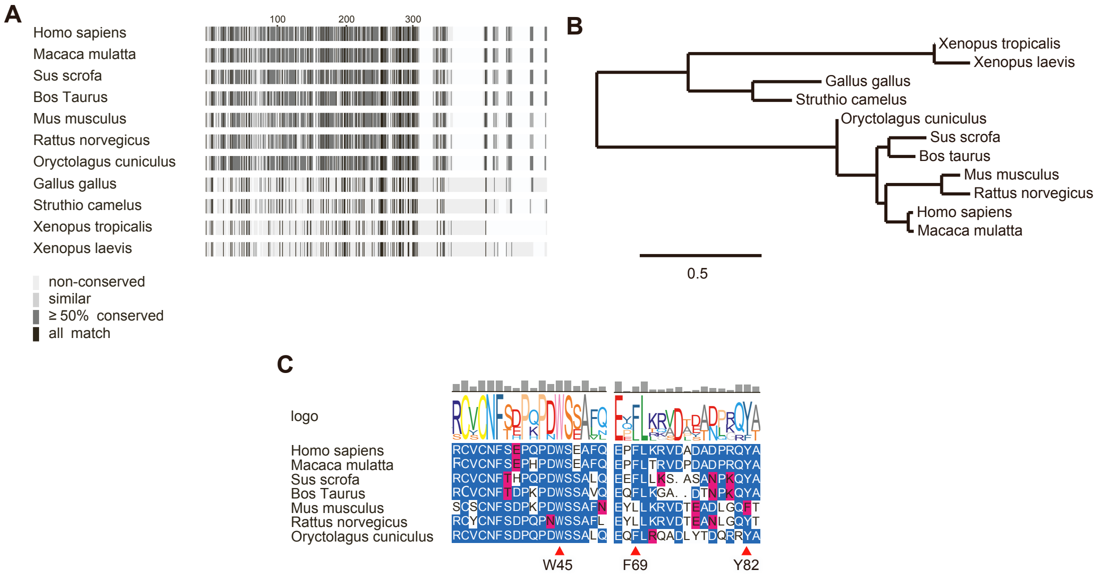

Figure S13 | Sequence conservation of CD14 in vertebrates. (A) Multiple sequence alignment of CD14 from various species represented as fingerprints with conserved sequence coloured in dark grey. (B) Phylogenetic tree of CD14 based on the sequence alignment built using PhyML and TreeDyn algorithms (www.phylogeny.fr). (C) Sequence alignment of N-terminal residues of CD14 coloured in blue for identical residues and magenta for similar residues. The degree of conservation is illustrated by the grey bars on top and the consensus sequence is shown in the middle as a WebLogo representation. The positions of residues that form cation-pi and staggered pi stacking interaction with peptide N-terminal arginine and histidine, respectively, are indicated by red triangles.

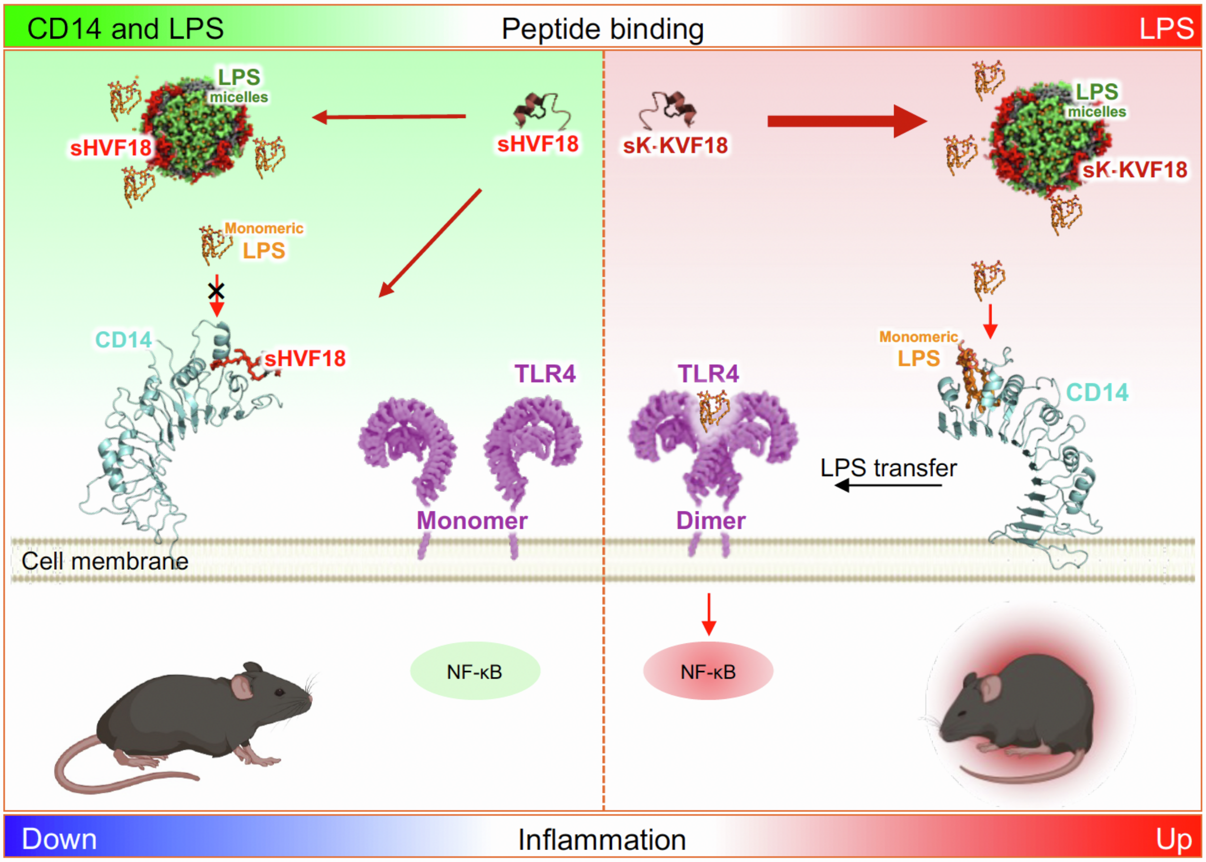

Figure S14 | Schematic figure illustrating how affinity of the peptide to LPS or CD14 and LPS affects inflammation in mice in CLP-induced sepsis. sHVF18, in addition to binding to LPS, also competes with LPS for the same site on CD14. This competition interferes with the CD14–LPS interaction, thereby hindering LPS transfer to TLR4, subsequent TLR4 dimerization, and downstream activation of inflammation through NF-κB. On the other hand, sK-KVF18, while binding strongly to LPS, does not bind to CD14 with the same affinity as LPS. As a result, free LPS after binding to CD14 is transferred to TLR4, enabling TLR4 dimerization and initiating inflammation through NF-κB.

A

Species	Accession number	F2		
		dN	dS	dN/dS
Macaca mulatta	NM_001104585.1	0.0219	0.1257	0.1742
Sus scrofa	NM_001122985.1	0.0846	0.4487	0.1885
Bos Taurus	NM_173877.1	0.0953	0.8445	0.1128
Mus musculus	NM_010168.4	0.1052	0.7744	0.1358
Rattus norvegicus	NM_022924.2	0.1159	0.8550	0.1356
Gallus gallus	NM_204605.2	0.2878	1.3932	0.2066
Struthio camelus	XM_009682749.1	0.2907	1.4866	0.1955
Xenopus tropicalis	NM_001015797.1	0.3247	2.5372	0.1280

B

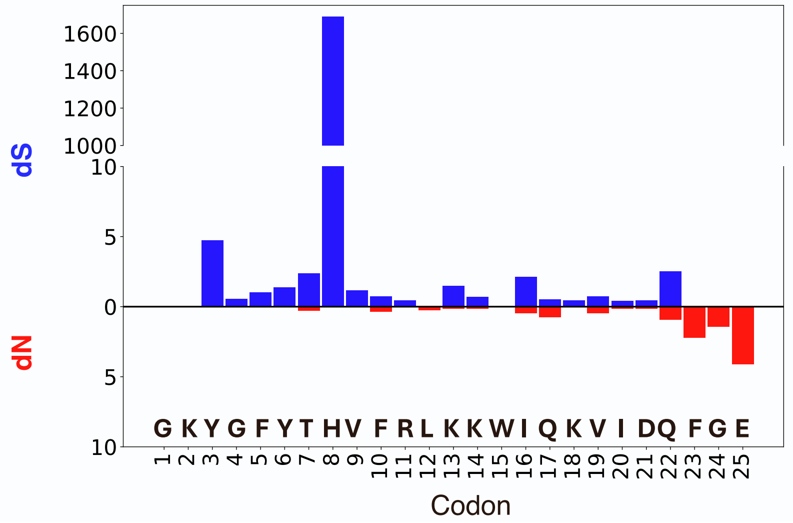

Figure S15 | Selection pressure on the F2 gene coding for thrombin. (A) Pairwise comparison of the full-length human F2 mRNA transcript sequence (GeneBank accession number: NM_000506.5) with other vertebrate sequences. The table shows the rate of non-synonymous substitution (dN), the rate of synonymous substitution (dS), and the ratio of dN/dS. Analysis was performed using the YN00 module in the PAMLX software. (B) Per codon maximum likelihood estimates of dS (blue) and dN (red) for the F2 transcript coding for the GKY25 peptide. Estimates above 10 are not shown. Resides coded by each codon in the human sequence are indicated. Analysis was performed using the Fixed Effects Likelihood (FEL) tool on Datamonkey webserver.
