# Supplementary material for: The conserved N-terminal histidine in an engineered peptide mediates sepsis treatment efficacy via dual binding to CD14 and LPS
Source: Mol Ther. 2025 Sep 23;34(1):407–22. doi: 10.1016/j.ymthe.2025.09.033 (PMC12925814; doi:10.1016/j.ymthe.2025.09.033)
Supplement: Document S2. Article plus supplemental information [file mmc2.pdf]

Molecular Therapy
Original Article

The conserved N-terminal histidine in an engineered peptide mediates sepsis treatment efficacy via dual binding to CD14 and LPS

Ganna Petruk,[1,7] Firdaus Samsudin,[2,7] Manoj Puthia,[1] Jitka Petrlova,[1,3,4] Peter J. Bond,[2,5] and Artur Schmidtchen[1,6]

[1]Division of Dermatology and Venereology, Department of Clinical Sciences, Lund University, 22241 Lund, Sweden; [2]Bioinformatics Institute (BII), Agency for Science, Technology and Research (A*STAR), 30 Biopolis Street, #07-01 Matrix, Singapore 138671, Republic of Singapore; [3]Department of Biomedical Science, Faculty of Health and Society, Malmö University, 20506 Malmö, Sweden; [4]Biofilms Research Centre for Biointerfaces, Malmö University, 20506 Malmö, Sweden; [5]Department of Biological Sciences, National University of Singapore, Singapore 117543, Republic of Singapore; [6]Dermatology, Skåne University Hospital, 22185 Lund, Sweden

Sepsis remains a major clinical challenge due to the limited efficacy of existing therapies in controlling excessive inflammation. The engineered stapled peptide sHVF18, derived from an evolutionarily conserved thrombin innate fold, binds both lipopolysaccharide (LPS) and the LPS-binding groove of CD14, enabling dual targeting of bacterial components and host immune signaling. To define structural prerequisites for this dual action, we combined evolutionary analysis, *in silico* modeling, and experimental methods. Substituting the N-terminal histidine with lysine (K) or arginine (R) improved solubility, reduced aggregation, and enhanced interactions with LPS. However, unexpectedly, K substitutions impaired CD14 binding, whereas R variants retained weaker affinity, possibly through cation-π interactions. The essential role of the evolutionarily conserved N-terminal histidine for CD14 interactions and therapeutic efficacy was demonstrated using LPS-induced shock and polymicrobial sepsis models. While the K variant exhibited superior efficacy in LPS-induced shock, its disrupted CD14 interactions rendered it ineffective in polymicrobial sepsis. In contrast, sHVF18, by engaging both LPS and CD14, effectively reduced inflammation and improved survival in polymicrobial sepsis. These findings highlight that targeting of both LPS and CD14 is essential for therapeutic efficacy, underscoring multivalency as a key principle for future sHVF18-based sepsis therapeutics.

INTRODUCTION

The development of peptide-based drugs has become a pivotal focus in pharmaceutical research,[1] highlighted by the US Food and Drug Administration's approval of 26 peptide drugs between 2016 and 2022, amid a broader landscape of 315 new drug approvals. With over 200 peptides currently in clinical development and an additional 600 in preclinical stages, interest in peptide therapeutics continues to grow.[2] An example of the success of peptide therapeutics is seen with GLP-1 agonists like semaglutide, which underscores the potential of peptides as drugs.[3] Peptides offer notable advantages over small molecules and larger proteins like antibodies, including greater target specificity, fewer off-target effects, and more predictable metabolic pathways, often resulting in non-toxic metabolites.[2]

Their susceptibility to enzymatic degradation remains a challenge, however. To overcome these limitations and expand their therapeutic potential, various structural modifications have been introduced. Peptide stapling via incorporation of side-chain covalent hydrocarbon bridges has shown promise in stabilizing peptide structures, improving proteolytic resistance, and enhancing bioavailability by extending plasma half-lives.[4–6] Despite these advances, no stapled peptide-based therapeutics have yet received regulatory approval.

So far, very few peptides have been approved for treating infectious diseases, even though peptide-based drugs have found applications in metabolic disorders, cancer, and cardiovascular diseases.[1] This is particularly concerning given that infectious diseases accounted for approximately 13.7 million deaths worldwide in 2019,[7] underscoring the urgent need for effective drug candidates. Moreover, current antibacterial treatments based on antibiotics predominantly target pathogens while neglecting the dysregulated inflammatory response and systemic complications like sepsis that accompany infections.

sHVF18 was designed to address the need for novel anti-infective and anti-inflammatory drugs for infective-inflammatory systemic indications. sHVF18 is a peptide-based mimetic of the active innate fold encoded in thrombin-derived C-terminal peptides (TCPs), which represents a new class of drugs inspired by nature's anti-infective mechanisms. TCPs, including TCP-25, have been widely studied for their dual ability to clear pathogen-associated molecular patterns like lipopolysaccharide (LPS) and regulate inflammation by targeting

Received 26 March 2025; accepted 19 September 2025;
https://doi.org/10.1016/j.ymthe.2025.09.033.

[7]These authors contributed equally

Correspondence: Ganna Petruk, Division of Dermatology and Venereology, Department of Clinical Sciences, Lund University, 22241 Lund, Sweden.
E-mail: ganna.petruk@med.lu.se

© 2025 The Author(s).
Published by Elsevier Inc. on behalf of The American Society of Gene and Cell Therapy.
This is an open access article under the CC BY license (http://creativecommons.org/licenses/by/4.0/).

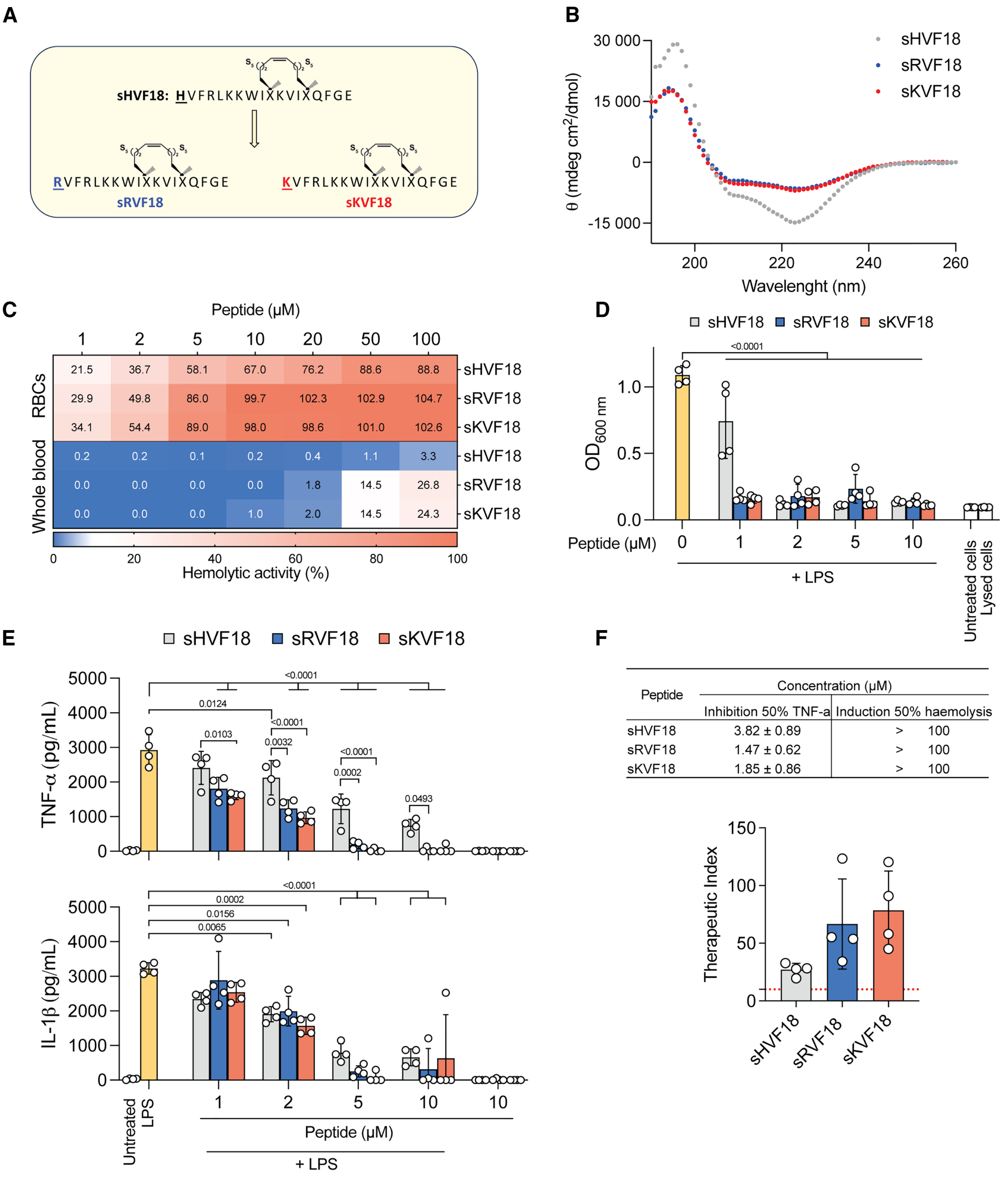

Figure 1. Functional analysis of new variants of sHVF18

(A) Illustration of sHVF18 and its arginine (R) and lysine (K) variant sequences.

(B) Circular dichroism spectra of sHVF18, sRVF18, and sKVF18 dissolved in 10 mM Tris at pH 7.4. Data are shown as means ± SDs of three different experiments (n = 3).

(C) The curves show the hemolytic activity of the peptides on erythrocytes (RBCs) or whole blood. Results are presented as means ± SDs of four different experiments (n = 4). For each experiment, blood from a different donor was used.

(D) NF-κB activation and cell viability in THP1-XBlue-CD14 reporter cells stimulated with 100 ng mL^{-1} *E. coli* LPS in the presence or absence of increasing concentrations of sHVF18 and its variants, 20 h poststimulation. Results are presented as means ± SDs of four experiments (n = 4).

(legend continued on next page)

CD14, a Toll-like receptor co-receptor.[8–12] TCP-25 is progressing in clinical development as a topical wound treatment.[13] However, it is prone to protease degradation, which is beneficial in wounds leading to the generation of bioactive fragments[10] and boosting endogenous host defense mechanisms but problematic for systemic use, requiring larger, more frequent doses and complicating clinical development. Therefore, through a combination of structure-based design, evolutionary insights, and molecular simulations, sHVF18 was engineered by stapling the C-terminal 18 residues of TCP-25 (HVFRLKKWIQ KVIDQFGE). This design enhanced proteolytic stability while improving affinity to CD14 and preserving high-affinity binding to LPS. The resulting peptide demonstrated significant therapeutic potential for systemic applications, showing improved efficacy in endotoxin shock models and increased survival rates in polymicrobial infection in mice.[14]

In this study, we focused on exploring the N-terminal sequence of sHVF18 to gain a deeper understanding of the structural prerequisites necessary for effective therapeutic interactions with LPS and CD14. Specifically, our aim was to evaluate how substituting the N-terminal histidine with arginine or lysine affects solubility and binding to LPS and CD14 and functional activity both *in vitro* and *in vivo*, to better guide future peptide optimization strategies.

RESULTS

Design strategy for new variants of sHVF18

Previous results have demonstrated that the protonation of the N-terminal histidine in TCPs enhances interactions with LPS. Additionally, increasing the overall charge of the peptide can improve solubility and, consequently, bioavailability. With the aim of preserving the internal CD14-interacting sequence, we sought to replace the N-terminal histidine in sHVF18 with charged residues. We therefore synthesized variants of sHVF18 (Figure 1A), replacing the N-terminal histidine with either arginine (sRVF18) or lysine (sKVF18). First, we confirmed the secondary structure of the peptides using circular dichroism. Regardless of the buffer system, neutral (Figure 1B) or acidic (Figure S1), all peptides exhibited an α-helical structure. Then, we compared the solubility of linear and stapled HVF18, as well as of the new variants of sHVF18, in buffers with neutral and acidic pH. As shown in Figures S1B and S1C, stapling increased the solubility of HVF18 at pH 7.4, since less peptide was detected in the pellet after centrifugation. Similar behavior was observed for the variants of sHVF18. As expected, all peptides were highly soluble at pH 5. We previously showed that the reduced solubility of TCPs at neutral pH, compared to acidic conditions, is attributable to oligomerization.[15] To investigate this for sHVF18 and its N-terminal variants, we used dynamic light scattering (DLS) to measure the hydrodynamic radius of peptide particles in solution. This approach allowed us to assess whether hydrocarbon stapling or substitution of the N-terminal histidine with arginine or lysine affects peptide aggregation or oligomerization. In agreement with our previous findings, the linear peptide showed greater aggregation/oligomerization propensity compared to the stapled peptides (Figure S1D), particularly at neutral pH.

Functional analysis of new variants of sHVF18

To understand the impact of replacing histidine with arginine or lysine on the hemolytic effect of the peptide, we exposed freshly isolated erythrocytes (red blood cells [RBCs]) and whole blood to increasing concentrations of sHVF18, sRVF18, and sKVF18. As shown in Figure 1C, the substitution of histidine resulted in increased hemolysis. Next, we investigated whether this substitution affected the anti-inflammatory activity of the peptide. To accomplish this, we stimulated THP1-XBlue-CD14 reporter cells with *Escherichia coli* LPS in the presence and absence of increasing concentrations of sHVF18, sRVF18, and sKVF18. The results indicate that the sRVF18 and sKVF18 variants inhibit LPS-induced nuclear factor κB (NF-κB) activity at a concentration of 1 μM (Figure 1D). To validate this finding under more physiological conditions, we tested the activity of these peptides in human blood stimulated with *E. coli* LPS. Significantly lower cytokine production was observed in blood stimulated with LPS in the presence of sRVF18 and sKVF18 compared to sHVF18 (Figure 1E). These findings translate into a higher therapeutic index (TI) for the sHVF18 variants compared to the parent peptide (Figure 1F), although all peptides demonstrate a TI greater than 10.

Based on these results, we decided to investigate whether adding additional arginine and lysine residues could further enhance the activity of the peptide. We found that the hemolytic activity on RBCs increased with the number of positively charged residues (Figure S2A). In a whole-blood experiment, however, one or two residues of arginine or lysine exhibited a similar hemolytic effect, while three or four residues drastically increased hemolysis (Figure S2B). Correspondingly, the anti-inflammatory activity of these peptides in THP1-XBlue-CD14 reporter cells stimulated with *E. coli* LPS (Figures S3A and S3B) was similar to the hemolysis data on RBCs (Figure S2A), showing a direct proportionality to the number of arginine and lysine residues. Similarly, in whole blood, the anti-inflammatory activity of these peptides (Figures S4A and S4B) correlated with hemolysis levels (Figure S2B). Specifically, up to two residues of arginine or lysine positively affected the anti-inflammatory activity of the peptide, while three and four residues exhibited lower activity at 1 and 2 μM. Based on these findings, we decided to exclude sRR- and sRRR-RVF18, as well as sKK- and sKKK-KVF18, from further analysis.

(E) Cytokine release from human blood stimulated with 100 ng mL^{-1} *E. coli* LPS in the presence or absence of increasing concentrations of sHVF18 and sVFR17, 24 h poststimulation. Results are presented as means ± SEMs of four experiments, each performed with blood from a different donor ($n = 4$).

(F) The table shows the concentration of peptides ± SD at which inhibition of 50% TNF-α in blood stimulated with LPS occurs and induces 50% hemolysis in whole blood. Data were obtained by plotting the percentage of hemolysis (shown in C) or TNF-α reduction (shown in E) with respect to the control as a function of peptide concentration. p values were determined using an ordinary two-way ANOVA followed by Tukey's multiple comparisons tests using GraphPad Prism software.

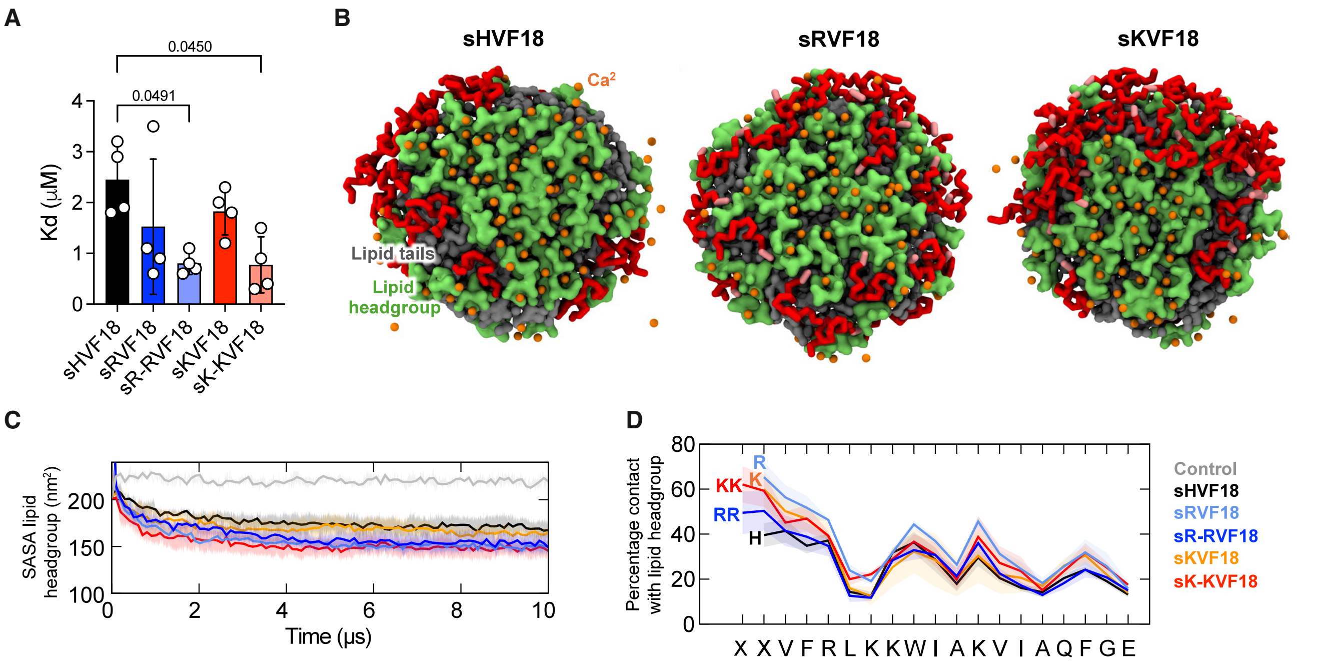

Figure 2. Histidine replacement at the N-terminal positively affects LPS binding

(A) The binding affinity of peptides to LPS-FITC were measured by MST. The K_D values were calculated from MST binding curves. The data are presented as means ± SDs of four different experiments (*n* = 4).

(B) CG simulations of peptides with lipid A aggregate. A small lipid A aggregate containing 61 lipids was simulated with 30 copies of sHVF18 or modified peptides positioned at least 2 nm away from the surface of the lipid aggregate. Three independent 10-μs coarse-grained (CG) simulations were performed. The figures show the final snapshots from one of the simulations for sHVF18, sRVF18, and sKVF18. Peptides are colored red, with the N-terminal arginine or lysine residues highlighted in pink. The lipids are shown in surface representation with the head group in green and tails in gray. Ca^{2+} ions are shown as orange spheres.

(C) Solvent-accessible surface area (SASA) of lipid head groups throughout the simulations with peptides and without peptides (gray). Thick lines show the average over three independent simulations, and the SD is depicted as the shaded areas. A probe radius of 0.26 nm was used for the calculation to represent the radius of CG water particles in the Martini force field.

(D) Percentage of contacts made by each residue of the peptide with lipid head groups. The cutoff distance for contact measurement is 0.6 nm.

Arginine and lysine substitutions reduce peptide aggregation

To understand the effects of N-terminal substitution on peptide aggregation, we performed coarse-grained (CG) molecular dynamics (MD) simulations of 10 copies of arginine- or lysine-substituted peptides in solution (details in materials and methods). We found substantially reduced peptide aggregation when the N-terminal histidine was replaced by these basic residues. Analysis of cluster sizes throughout the trajectories revealed that the original sHVF18 peptide formed a large singular aggregate within the first 2 μs of the simulations and maintained this peptide cluster for the remainder of the simulations (Figures S5A and S5B). In contrast, none of the arginine- or lysine-substituted peptides clustered into one aggregate by the end of the 5-μs simulations. They formed smaller clusters, the largest of which were composed of 4–8 peptides, and dynamically disaggregated and aggregated throughout the trajectories. Contact analysis showed that the N-terminal histidine had a significantly higher propensity to interact with other peptides compared to arginine and lysine (Figure S5C). This suggests that the positively charged arginine or lysine residue at the N terminus prefers to interact with solvents rather than with other peptides, contributing to the reduced peptide aggregation in solution. Our simulations are thus in good agreement with results from the peptide solubility experiments presented in Figure S1.

Arginine and lysine substitutions improve interaction with LPS

Given the increased net positive charge of these peptides at neutral pH and their reduced tendency to aggregate, we postulated that they would exhibit increased LPS binding. Our microscale thermophoresis (MST) results showed that substitution of N-terminal histidine with arginine or lysine resulted in lower K_D, particularly with two residues (i.e., in sR-RVF18 and sK-KVF18 variants) (Figure 2A). More specifically, we determined the following K_D values: 2.45 ± 0.70 μM for sHVF18, 1.53 ± 1.33 μM for sRVF18, 0.80 ± 0.22 μM for sR-RVF18, 1.83 ± 0.46 μM for sKVF18, and 0.78 ± 0.55 μM for sK-KVF18. To elucidate the underlying molecular mechanism of the increased affinity for these peptides to LPS, we performed CG MD simulations of sHVF18 and modified peptides with a lipid A aggregate. Lipid A is the bioactive lipid component of LPS, which we have shown in previous studies to be the primary binding site for TCPs.[9,14] We found that all 30 copies of these peptides adsorbed onto the surface of a lipid A

aggregate within the first 2 μs of the simulations and remained in contact throughout the rest of the simulations (Figures 2B and S6A). Visual inspection of CG MD simulations indicated that peptides with N-terminal arginine or lysine made more contacts with the lipid head group compared to sHVF18.

We next measured solvent-accessible surface area (SASA) of the lipid head group (Figure 2C) and lipid tails (Figure S6B) separately to quantify these interactions. As expected, SASAs for both parts of the lipid were reduced throughout the simulations compared to control simulations without peptides due to peptide adsorption. Interestingly, we observed significantly lower lipid head group SASA for peptides with two arginine or lysine substitutions compared to the original sHVF18. Contact analysis revealed that the N-terminal arginine or lysine residues made more contacts with the lipid head group compared to histidine (Figure 2D). The percentage of contacts with lipid tails was similar for all peptides (Figure S6C). Divalent cations such as Ca^{2+} ions play an important role in intercalating phosphate groups in LPS head groups. We found that peptides with two N-terminal arginine or lysine residues displaced more Ca^{2+} ions than sHVF18, corroborating the more pronounced interactions made by these residues with the lipid head group (Figure S6D). As both arginine and lysine are positively charged, it is expected that they will form better ionic interactions with the negatively charged phosphate groups on LPS compared to histidine. Additionally, having two of these residues, such as in sR-RVF18 and sK-KVF18, will increase the possible number of contacts with the lipid head group. Collectively, our simulations demonstrated that the higher binding affinities of lysine- and arginine-substituted peptides by our MST experiments originate from the improved interactions made by these peptides with LPS head groups.

Arginine-substituted peptides bind better to CD14 than lysine counterparts

Next, we investigated the effects of arginine and lysine substitutions on CD14 binding. We measured the binding affinity of all peptides to CD14 in solution using MST. Surprisingly, either replacement resulted in higher K_D values: 2.58 ± 1.24 μM for sHVF18, 4.30 ± 2.59 μM for sRVF18, 17.48 ± 3.42 μM for sR-RVF18, 37.37 ± 14.79 μM for sKVF18, and 144.02 ± 43.04 μM for sK-KVF18 (Figure 3A). Additionally, peptides with two residues of arginine or lysine exhibited higher K_D values compared to those with just one residue. Notably, arginine substitution had a less pronounced effect on KD compared to lysine.

To understand the underlying molecular mechanism behind the different binding affinities, we modeled the binding of CD14 to the arginine- and lysine-substitute peptides. Using the CD14-sHVF18 complex structure from our previous study as a template,[14] we replaced the N-terminal histidine with either one or two arginine or lysine residues and performed microsecond timescale atomic-resolution simulations. All peptide variants remained bound to the binding groove of CD14, although the positions of the N terminus shifted compared to the initial structure, indicating that these peptides adopted different interactions with CD14 residues compared to histidine (Figures 3B and S7A). We measured root-mean-square deviation (RMSD) of the whole peptide after a least-squares fit to the initial structure of the complex and found that the singly substituted peptides sRVF18 and sKVF18 showed slightly higher RMSDs, although within the error bars compared to sHVF18 (Figure S7B). The sR-RVF18 peptides exhibited much lower RMSD compared to sK-KVF18, suggesting significantly more stable binding of the arginine-substituted peptide compared to the lysine counterpart. Congruently, our MST measurements also demonstrated significantly lower K_D values for arginine-substituted peptides, indicating higher CD14 binding affinity (Figure 3A).

The different binding stability of arginine- and lysine-substituted peptides could arise from interacting with different residues in the CD14 binding site. Contact analysis revealed that the positively charged ammonium group in the N-terminal lysine of sKVF18 and sK-KVF18 interacted mostly with negatively charged residues scattered around the CD14 binding groove (Figure S7C). Conversely, the guanidinium group of N-terminal arginine in sRVF18 and sR-RVF18 made contacts with more diverse residues, including aromatic residues such as W45, F69, and Y82 (Figure 3C). Indeed, simulation trajectories showed that the N-terminal arginine in sRVF18 can form a cation-π interaction with W45 and a hydrogen bond with Y82 simultaneously (Figure 3D). Similarly, the arginine residues in the N terminus of sR-RVF18 formed cation-π interactions with F69 and Y82. Distance measurements suggested that some of these interactions were stable and lasted throughout most of the trajectories (Figures S8A and S8C). However, similar distance measurements with the ammonium group of the N-terminal lysine revealed that these cation-π interactions and hydrogen bond were absent in sKVF18 and sK-KVF18 (Figures S8B and S8D). To confirm the role of aromatic residues on CD14 in the binding of arginine peptide variants, we mutated residues W45, F69, and Y82 to alanine and performed all-atom MD simulations with sRVF18. RMSD calculations indicated that peptide binding to the mutant CD14 is less stable than to the wild type (Figure S9A). Cluster analysis showed that the side chain of the N-terminal arginine was no longer able to form cation-π interaction with mutated residues W45A and Y82A on CD14; instead, it formed a hydrogen bond with the backbone carbonyl group of F69A (Figure S9B). Indeed, a comparison of distance measurements between side chains highlights the loss of contacts between arginine and W45A and Y82A (Figure S9C), which could explain the reduced stability of peptide binding. Hence, our simulations of peptide-bound mutant CD14 further corroborated the role of aromatic residues in stabilizing the binding of arginine-substituted peptides.

Finally, we compared the interactions of these modified peptides to the original sHVF18 (Figure S10A). Overall, the N-terminal residues of these peptides made contact with different residues on CD14; however, some overlaps exist—for example, both sHVF18 and sRVF18 made significant interactions with hydrophobic residues such as W45 and F69 (Figure S10B). Comparing all contacts made with CD14, we found a higher correlation between the original

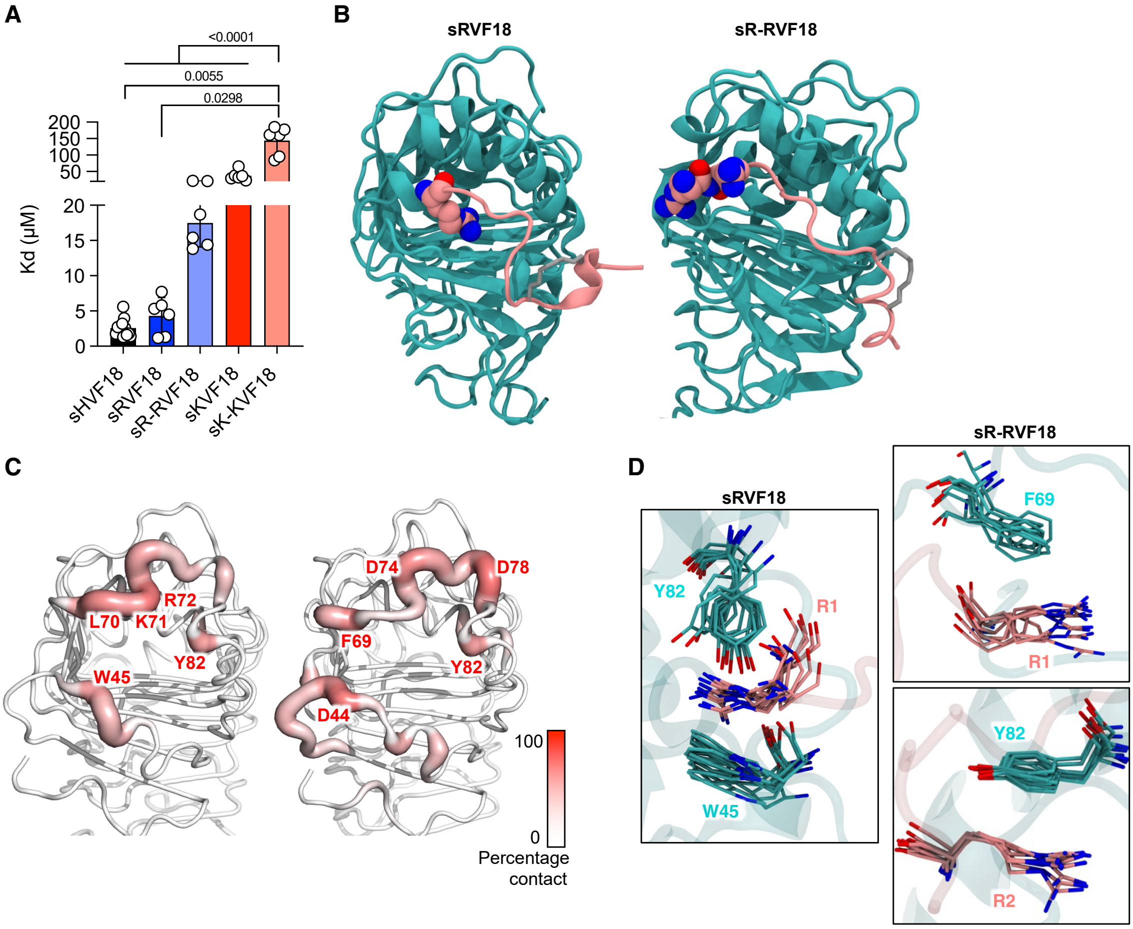

Figure 3. Histidine replacement at the N-terminal negatively affects CD14 binding

(A) The binding affinity of peptides to labeled CD14 was measured by MST. The K_D values were calculated from MST binding curves. The data are presented as means ± SDs of at least six different experiments ($n \geq 6$). Note that a break in the *y* axis was inserted to improve the resolution of smaller K_D values.
(B) All-atom MD simulations of CD14 complexed with arginine-substituted peptides. The N-terminal histidine residue was mutated to either one or two arginine residues and three independent 1-μs simulations were performed. The figures show CD14 (cyan) and peptide (pink) with the N-terminal arginine residue(s) depicted in van der Waals representation, taken from the central structure of the top cluster from a cluster analysis performed on concatenated trajectories.
(C) Average percentage of contacts made by CD14 residues with the guanidinium group of arginine at the N terminus of modified peptides from three simulations. The top five residues that made the most contacts are labeled.
(D) Overlaid snapshots taken every 50 ns from parts of the simulations showing cation-π and polar interactions between the N-terminal arginine residue(s) on the peptide (pink) and aromatic residues on CD14 (cyan).

sHVF18 peptide and sRVF18 (ρ = 0.40) compared to sKVF18 (ρ = 0.12; Figure S10C). Simulation snapshots of sHVF18 bound to CD14 showed π-π stacking interaction between the side chain of N-terminal histidine on the peptide with residues W45 and F69 on CD14 (Figure S10D). These CD14 residues also formed cation-π interactions with the N-terminal arginine on sRVF18 (Figure 3D), further corroborating that the histidine and arginine N termini share more common interactions with CD14 than lysine. Collectively, our simulations revealed that the arginine-substituted peptides can tolerate the hydrophobic binding site of CD14 better than their lysine counterparts due to the ability to form cation-π interactions and hydrogen bonds with surrounding residues.

The effect of deleting histidine on sHVF18 toward peptide function

The role of N-terminal histidine in CD14 binding is not well understood. To elucidate this, we first measured how removing the histidine residue from the peptide sequence affected its binding affinity to LPS and CD14. As shown in Figure 4A, the variant without histidine exhibited higher K_D values in both cases. The removal of histidine had a more pronounced effect on the K_d for CD14 compared to LPS. The affinity of sHVF18 to CD14 was 2.70 ± 1.59 μM, compared to 33.52 ± 12.83 μM for sVFR17. In contrast, the affinity of sHVF18 to LPS was 2.45 ± 0.70 μM, while that of sVFR17 was 8.45 ± 4.36 μM. The reduced affinity of sVFR17 for LPS and CD14 led to a loss of its

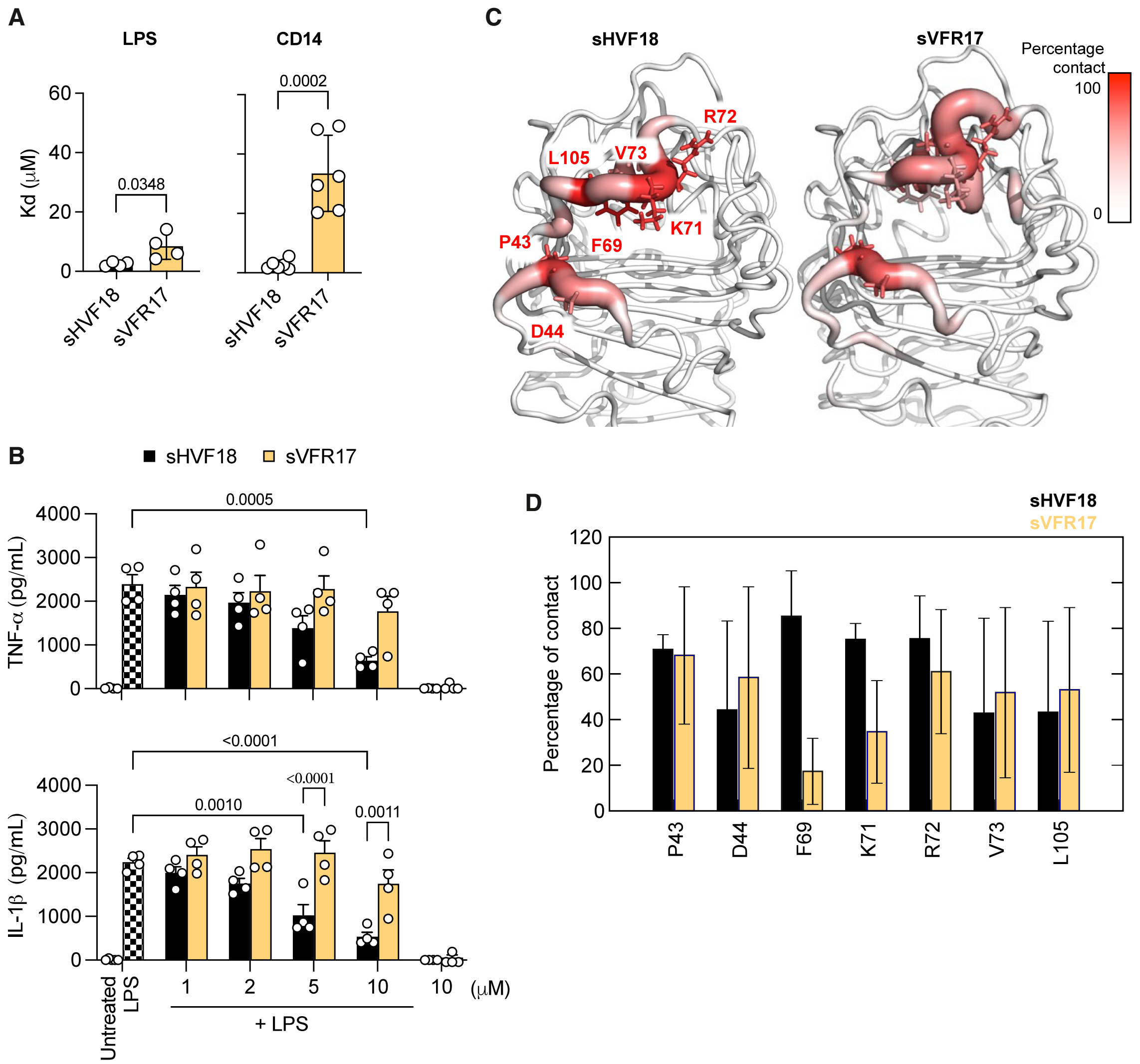

Figure 4. The effect of deleting histidine on sHVF18 toward peptide function

(A) The binding affinity of peptides to LPS or CD14 was measured by MST. The K_D values were calculated from MST binding curves. The data are presented as means ± SDs of at least four different experiments ($n \geq 4$).

(B) TNF-α and IL-1β release from human blood stimulated with 100 ng mL^{-1} *E. coli* LPS in the presence or absence of increasing concentrations of sHVF18 and sVFR17, 24 h poststimulation. Results are presented as means ± SEMs of four experiments, each performed with the blood from a different donor ($n = 4$). p values were determined using an ordinary two-way ANOVA followed by Tukey's multiple comparisons tests using GraphPad Prism software.

(C) All-atom simulations of CD14 complexed with sHVF18 and sVFR17. The N-terminal histidine residue on sHVF18 was deleted from the representative structure of sHVF18-CD14 complex, and three independent 1-μs simulations were performed. The heatmaps show CD14 residues that made most contacts with the N-terminal H1 of sHVF18 and V1 of sVFR17 during the simulations. (D) Comparison of average percentage of contacts made by these residues, with the SDs from three simulations shown as error bars.

anti-inflammatory activity when tested in blood stimulated with *E. coli* LPS (Figure 4B).

Next, we modeled the binding of CD14 to sVFR17 using the structure of CD14-sHVF18 complex from our previous study as the template. All-atom simulations showed that while the peptide remained bound to the binding site on CD14, the N terminus shifted compared to the initial position (Figure S11A). RMSD calculations also indicated increased dynamics of the N terminus compared to sHVF18 (Figure S11B). Contact analysis revealed that the truncated peptide made fewer interactions with key CD14 residues that bind to the N-terminal histidine in sHVF18 (Figures 4C and 4D). Hence, the N-terminal histidine could play a crucial role in the orientation of the N terminus of the peptide during CD14 binding.

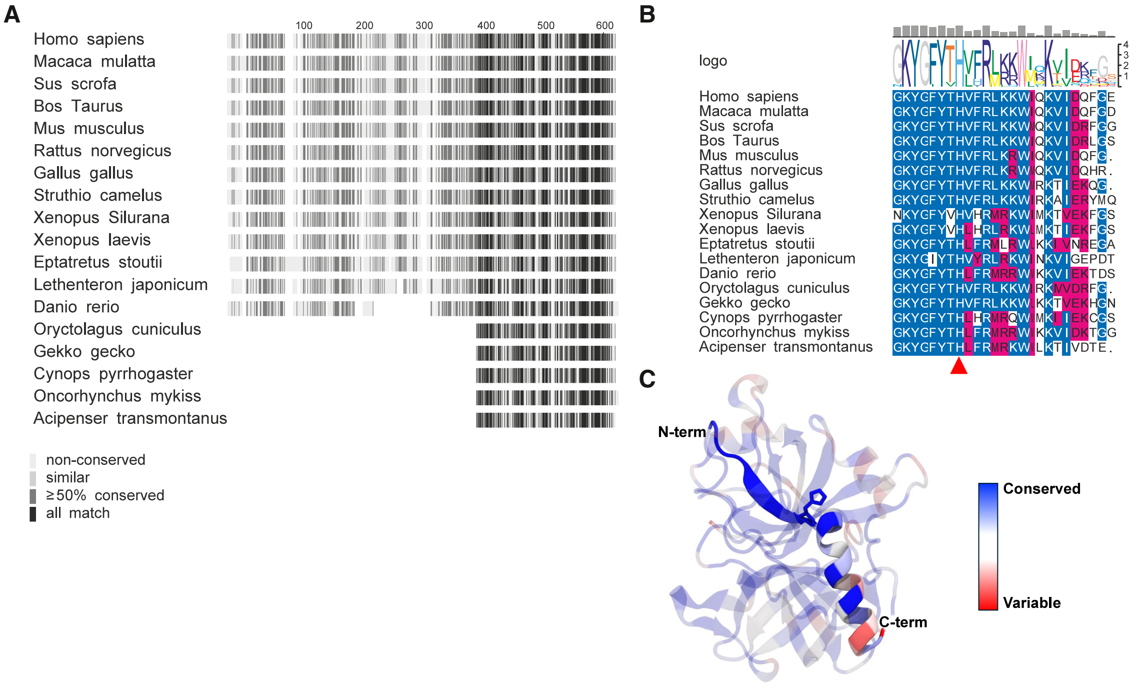

Figure 5. Histidine is conserved in thrombin sequences across vertebrates

(A) Multiple sequence alignment of thrombin from various species represented as fingerprints, with conserved sequence colored in dark gray.

(B) Sequence alignment of the last 25 residues from the C terminus (TCP25) colored blue for identical residues and magenta for similar residues. The degree of conservation is illustrated by the gray bars on top, and the consensus sequence is shown in the center as a WebLogo representation. The position of the N-terminal histidine residue in HVF18 peptide is indicated by the red triangle.

(C) The degree of conservation is mapped to the crystal structure of human thrombin (PDB: 3U69) and colored from red (variable) to blue (conserved). TCP25 is highlighted and the rest of the protein is shown in transparent representation. N-terminal histidine residue in HVF18 peptide is shown in stick representation.

Histidine is conserved in thrombin but not in homologous proteins

Given the impact of the N-terminal histidine in sHVF18 on CD14 binding, we decided to investigate the evolutionary conservation of this histidine residue in thrombin. A multiple sequence alignment of prothrombin from various vertebrates showed a high degree of sequence conservation in the C-terminal end of the protein (Figure 5A). Further inspection of the histidine (H605 in full-length prothrombin sequence including signal peptide) revealed that the residue is universally conserved (Figure 5B). Apart from the histidine, the TCP-25 sequence, in general, is highly conserved on the N-terminal end, whereas the C-terminal region is less conserved (Figure 5C).

We then investigated whether the histidine residue is found in other related proteins. We performed a BLAST search for homologous proteins in humans using the sequence of prothrombin as a query sequence. As expected, we found several S1 serine endopeptidases with similar sequences. A multiple sequence alignment of these proteins with prothrombin indicated several conserved regions in the C-terminal end of the proteins (Figure S12A). Phylogenetic tree analysis suggested that prothrombin is most similar to anticoagulant protein C and several coagulation factors (Figure S12B). Thus, we performed a multiple sequence alignment of each of these similar proteins using vertebrate sequences to generate a consensus sequence for each protein (Figure S12C). Intriguingly, these consensus sequences showed that only prothrombin has a histidine residue at the H605 position (red triangle in Figure S12C), while other homologous proteins have either lysine or arginine. Notably, threonine was a dominant amino acid preceding histidine (Figures 5B and S12C).

CD14 residues that interact with histidine and arginine are conserved

Our simulations showed that the binding of the sRVF18 and sHVF18 N-terminal end to CD14 is stabilized by several aromatic residues via cation-π or π-stacking interactions (Figures 3D and S10D). We examined the evolutionary conservation of these residues in CD14 by performing a multiple sequence alignment using sequences from vertebrates (Figure S13A). The N-terminal region of CD14, which corresponds to the LPS and TCP binding sites, is highly conserved in a group of vertebrates, including mouse, rat, and pig. However, the sequence alignment showed a large divergence in certain vertebrates like chicken and camel. This is corroborated by a phylogenetic tree analysis which displayed an early bifurcation

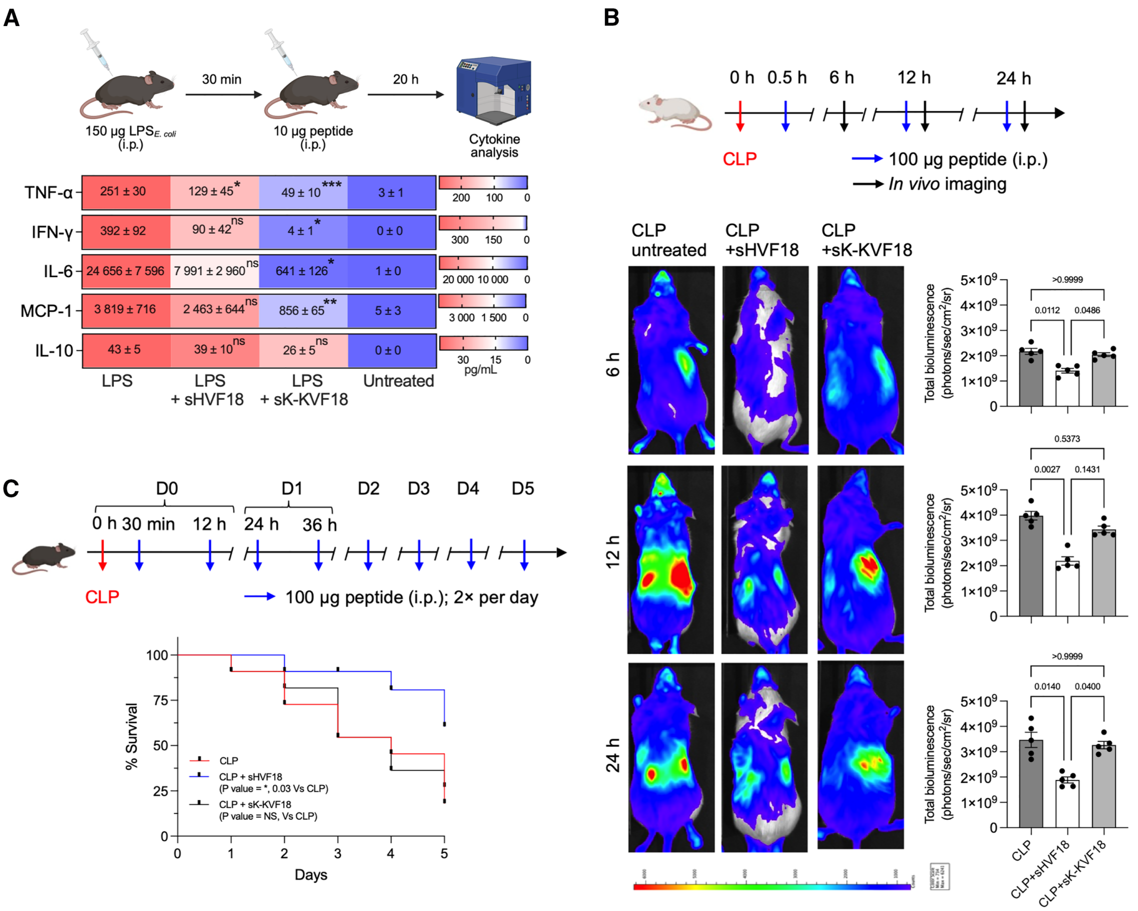

Figure 6. *In vivo* implication of histidine replacement for peptide activity

(A) Effects in mouse endotoxic shock model. (Top) Schematic overview of the experimental setup. C57BL/6 mice were stimulated intraperitoneally (i.p.) with 150 μg *E. coli* LPS. After 30 min, 10 μg of peptide was injected i.p. into the mice. Cytokine release in plasma was quantified by flow cytometry 20 h post-LPS stimulation. (Bottom) Heatmap showing cytokine release in mice following endotoxin shock. Cytokines were detected using flow cytometry. Colors represent mean values in pg mL^{-1}, and each box displays mean ± SEM.

(B and C) (Top) Schematic overview of the experimental setup of the polymicrobial sepsis model, followed by *in vivo* imaging (B) or survival (C). Polymicrobial sepsis in BALB/c tg(NF-κB-RE-Luc)-Xen reporter mice (B) or C57BL/6 (C) was induced by subjecting mice to the CLP procedure. At 30 min, the mice were i.p. treated twice daily with 100 μg peptide (in 100 μL water) for different lengths of time. The first dosage of peptide was given 30 min after the CLP procedure. The control group was given 100 μL water i.p. (B) Noninvasive *in vivo* bioimaging of NF-κB reporter gene expression was performed using the IVIS Spectrum system. Representative images show bioluminescence at 6, 12, and 24 h after CLP procedure. A bar chart shows measured bioluminescence intensity emitted from these reporter mice. Data are presented as means ± SEMs (n = 5). The p values were determined using a nonparametric test followed by Dunn's multiple comparisons test. (C) Kaplan-Meier survival curve of the percentage survival within 5 days after CLP. The p values were determined using the Kaplan-Meier method (n = 11 mice/group). Images of mice and cell membranes were obtained from BioRender.com.

between these two groups of vertebrates (Figure S13B). Thus, we focused our analysis on the group of vertebrates that share the same CD14 lineage with humans (Figure S13C). We found that residue W45, which interacted with both N-terminal histidine and arginine in our simulations, is conserved across all of these vertebrates. Residue F69 is conserved in most vertebrates, except in mouse and rat, which have a leucine substitution at the same position. Residue Y82, which interacted primarily with the N-terminal arginine, is also conserved, except in mice with a phenylalanine substitution; however, this residue will still be able to form a cation-π interaction. Overall, our analysis indicated that CD14 residues that form key interactions with the N-terminal histidine and arginine residues in TCPs are highly conserved across related vertebrates.

Efficacy in a polymicrobial sepsis model depends on CD14 binding

To highlight the importance of CD14 binding for a potential systemic drug, we compared the activity of sHVF18 and sK-KVF18 in two mouse models: endotoxin shock and polymicrobial sepsis. Consistent with our *in vitro* data, the stronger binding of LPS to sK-KVF18 compared to sHVF18 resulted in more effective mitigation of the LPS-induced cytokine storm (Figure 6A). However, in

the more complex context of polymicrobial sepsis, sK-KVF18 failed to reduce inflammation, as shown by imaging NF-κB reporter mice (Figure 6B), resulting in a lower survival rate (Figure 6C).

DISCUSSION

In drug development, understanding the structure-activity relationships is essential for optimizing the therapeutic efficacy of lead compounds. After identifying a lead compound such as sHVF18,[14] preclinical development focuses on refining its structure to maximize activity while ensuring favorable formulation properties. Our study builds on previous findings demonstrating the dual affinities of sHVF18 for CD14 and LPS, which are critical for its anti-inflammatory effects. By examining the structural requirements for these interactions—particularly the pivotal role of the N-terminal histidine in CD14 binding and its impact on therapeutic activity *in vivo*—we provide valuable insights for future peptide drug design. These findings emphasize the challenge of balancing CD14 binding capacity and LPS interactions with bioavailability through structural modifications.

Using sHVF18 as a template, we designed new peptides in which the N-terminal histidine was replaced with one to four residues of arginine or lysine, aiming to enhance solubility without compromising key functions like LPS and CD14 binding. Through hemolysis assays, we found that sHVF18 was less hemolytic than its arginine or lysine variants, with hemolysis increasing significantly in variants containing three or four residues of arginine or lysine. Notably, these variants also displayed reduced anti-inflammatory effects, likely due to the additional positive charges increasing non-specific binding, as seen in the elevated hemolysis. These data indicate that while replacing histidine with arginine or lysine residues improved certain properties, careful optimization of residue number is critical for balancing efficacy and safety.

Histidine acquires a positive charge only at acidic pH, while arginine and lysine carry positive charges under most physiological conditions.[16] Since sHVF18-LPS binding is partly driven by electrostatic interactions with the LPS head group,[14] we expected improved binding with the arginine and lysine variants. This hypothesis was confirmed through MST affinity measurements and *in silico* CG MD simulations, which showed that replacing histidine with one or two arginine or lysine residues reduced peptide aggregation and improved anti-inflammatory activity.

We next explored how CD14 binding was affected by these substitutions. Arginine-substituted peptides exhibited better CD14 binding than lysine variants, although still weaker than the original sHVF18. The lower affinity of lysine-substituted peptides likely stems from their positively charged side chains, which may not interact favorably with the hydrophobic CD14 binding pocket. Instead, lysine appeared to interact with negatively charged residues on CD14, suggesting that these variants act more as LPS scavengers rather than directly blocking CD14. In contrast, arginine variants interacted more effectively with hydrophobic residues such as W45, Y82, and F69 on CD14, forming cation-π interactions and hydrogen bonds, consistent with findings by Gallivan and Dougherty.[17] This enhanced interaction likely explains the lower K_D values observed for arginine variants in MST experiments. The critical role of histidine in CD14 binding was further highlighted by comparing sHVF18 with a variant lacking the N-terminal histidine. While histidine removal had little impact on LPS binding, it significantly reduced CD14 binding. This effect is likely due to the unique ability of histidine to engage in various interactions depending on its protonation state.[18] For example, we previously demonstrated that endogenous HVF18 binds CD14 more effectively at neutral pH than in acidic conditions,[19] underscoring the versatility and importance of histidine in peptide-CD14 interactions.

The negative implications of reduced CD14 binding capacity *in vivo*, despite enhanced LPS binding, were demonstrated as a proof of concept by comparing the activity of sHVF18 and sK-KVF18 in two different mouse models. While sK-KVF18 counteracted LPS-induced inflammation more effectively than sHVF18, it failed to retain its activity in the more complex context of polymicrobial sepsis. These findings highlight the critical importance of the dual interaction with both CD14 and LPS for effective downregulation of inflammation and improved survival in complex inflammatory conditions (Figure S14). To better understand why sHVF18 outperforms sK-KVF18 in this context, future studies should also assess the potential intrinsic antimicrobial activities of both peptides. For example, comparative evaluation in a monomicrobial sepsis model, such as *E. coli* infection, could help delineate their relative contributions to antimicrobial activity versus immunomodulatory effects.

An intriguing question is what structural and functional pressures have driven the high evolutionary conservation of the N-terminal histidine in the HVF18 sequence. This residue likely plays a critical role in maintaining the ability of the peptide to interact with key targets. From the perspective of whole thrombin, histidine often plays a key role in enzyme catalytic reactions.[20] Inspecting closely the catalytic site of thrombin, it emerges that histidine cannot be effectively replaced by other amino acids like arginine or lysine, as it is involved in proton transfer during cleavage of peptide bonds.[21] Indeed, with its imidazole side chain of pKa around 6, histidine can act as both a proton donor and acceptor across a range of pH levels found in the blood and tissues. This characteristic is not that important for thrombin homologs, such as coagulation factor VII, as it works at neutral pH found in blood and is not exposed to such a variety of pH conditions as thrombin.[22] Thus, this might be the explanation as to why histidine is conserved in thrombin sequences across vertebrates but is replaced by arginine or lysine in its homologs. In addition, histidine, through hydrogen bonds and other electrostatic interactions, stabilizes the enzyme.[18] Given the central role of thrombin in preventing excessive bleeding and enabling wound healing after trauma, it becomes clear that the stability of this enzyme is essential. Furthermore, considering the multifunctionality of thrombin, such as pro-/anticoagulant and pro-/anti-inflammatory roles,[23,24] and the fact that histidine is universally conserved in

thrombin but not in other homologous proteins raises questions about the specific importance of this residue for the function of thrombin. Indeed, genetic variations resulting in the replacement of this histidine with an arginine residue have been associated with prothrombin deficiency, a rare congenital bleeding disorder,[25,26] indicating its importance in thrombin primary function as a pro-/anticoagulant.

From the host defense perspective, thrombin undergoes enzymatic cleavage by neutrophil elastase or other bacterial enzymes, releasing a pool of TCPs.[8,27] These peptides, depending on their length, perform different tasks, such as antimicrobial action, clearance, and anti-inflammatory effects.[8,9,27–34] We speculate that another reason histidine is found in all TCPs is its involvement in CD14 binding. Indeed, the N-terminal region of CD14, which corresponds to the LPS and TCP binding site, is highly conserved in a group of vertebrates, including mice, rats, and pigs, particularly the amino acids involved in histidine binding. Finally, the histidine is not only preserved but conserved over time. For example, when we subjected the F2 gene coding for thrombin to evolutionary analysis, we observed that the codon coding for the histidine residue was by far the most conserved in the TCP sequence, as indicated by the high rate of synonymous substitution (Figure S15). The evolutionary adaptation that allows thrombin to serve as both a protease and a source of bioactive peptides demonstrates a sophisticated strategy where a single protein can evolve multiple functions. This duality not only reflects the adaptability of thrombin in responding to physiological demands but also emphasizes that use of such evolutionary clues could benefit today's drug development of sHVF18 and its variants.

Moreover, assuming that elastase has a conserved proteolytic specificity in vertebrates, whereby it cleaves at the C terminus of small uncharged amino acids (e.g., V, I, A, T),[35] our sequence alignment showed that a cleavage site N-terminal of the HVF18 sequence is universally conserved (Figure 5B); hence, hypothetically, during evolution, these vertebrates may have acquired a mechanism to produce TCPs during inflammation.

In summary, we have disclosed here a previously unknown importance of N-terminal histidine in the engineered sHVF18 peptide. Its replacement with one or two arginine or lysine residues showed better anti-inflammatory activity and lower aggregation, albeit with a lower CD14 binding affinity. This shifted the dual-action mechanism of the peptide toward primarily LPS scavenging. The observation that histidine is highly conserved in thrombin among vertebrates, along with its involvement in CD14 binding, underscores its functional significance. From a drug design and structure-function perspective, the role of the N-terminal histidine is a critical determinant of CD14 interaction and should be carefully considered in future development efforts. sHVF18 represents a promising lead compound for the treatment of severe infections in which both endotoxin neutralization and CD14 targeting are required. Structural modifications that retain the N-terminal histidine while incorporating additional features, such as lysine and/or arginine residues, may enhance solubility and modulate key functionalities without compromising CD14 binding. Moreover, the increased LPS-binding capacity observed in arginine- and lysine-containing variants may be leveraged in future studies focused on Gram-negative infections, such as *E. coli* sepsis.

MATERIALS AND METHODS

Peptides and proteins

The peptides HVF18 (HVFRLKKWIQKVIDQFGE), sHVF18 (HVFRLKKWIXKVIXQFGE), sVFR17 (VFRLKKWIXKVIXQFGE), sKVF18 (KVFRLKKWIXKVIXQFGE), sK-KVF18, sKK-KVF18, sKKK-KVF18, sRVF18 (RVFRLKKWIXKVIXQFGE), sR-RVF18, sRR-RVF18, and sRRR-RVF18 were synthesized and purified by AmbioPharm (USA) as reported in Petruk et al.[14] Briefly, during standard 9-fluorenylmethyloxycarbonyl solid-phase peptide synthesis, an olefin-bearing (*S*)-2-(4′-pentenyl)-alanine was inserted at specified locations in the respective peptide sequences (denoted by X) to obtain hydrocarbon stapled peptides. The product peptides were purified by reverse phase-high-performance liquid chromatography and provided as acetate salt. The purity was confirmed with MALDI-TOF mass spectrometry (>95%). Human His-tag CD14 (CD14) was produced in insect cells and purified, as reported previously.[36]

Ethics statement

Venous blood was collected from healthy donors after written informed consent was obtained. After collection, whole blood was used immediately for the experiments. The use of blood was approved by the ethics committee at Lund University, Lund, Sweden (permit no. DNR2015/801). All experiments on mice were performed according to Swedish Animal Welfare Act SFS 1988:534 and were approved by the Animal Ethics Committee of Malmö/Lund, Sweden (permit nos. M8871-19 and 16542-21). All animals had free access to water and chow and were housed on a 12-h light/12-h dark cycle in the Innovive IVC Rodent Caging System. The room temperature was kept between 19°C and 23°C and the humidity at 55% ± 10%.

Circular dichroism

Circular dichroism was used to assess the secondary structure of sHVF18 and its arginine and lysine variants. The peptides were diluted to 10 μM in 10 mM Tris at pH 7.4 or 10 mM NaOAc at pH 5.0. The spectra were acquired on a Jasco J-810 spectropolarimeter (Jasco, Japan) equipped with a Jasco CDF-426S Peltier set to 20°C, between 190 and 260 nm (scan speed: 20 nm min^{-1}) as an average of 3 measurements. A 0.2-cm quartz cuvette (Hellma GmbH, Germany) was used. The baseline (10 mM Tris at pH 7.4 or 10 mM NaOAc at pH 5.0) was subtracted from each spectrum, and the final signal was converted to mean residue ellipticity, θ (mdeg cm^2 $dmol^{-1}$), as reported by Morrissett et al.[37] The results are expressed as mean ± SD obtained from three independent experiments.

Solubility study

Peptides (HVF18, sHVF18, sKVF18, and sRVF18) were resuspended in 10 mM Tris at pH 7.4 or in 10 mM NaOAc at pH 5 to a final concentration of 1 mM, immediately before measuring the concentration using a NanoDrop (DeNovix DS-11, USA). Then, 30 μL of each sample were centrifuged at 14,000 × *g* for 20 min. The supernatant was transferred in a new tube and the concentration measured again by NanoDrop. The results are expressed as mean ± SD obtained from three independent experiments.

SDS-PAGE

Pellets and supernatants obtained in solubility assay were used for this experiment. Pellets were resuspended in 10 μL of the same buffer. We loaded 10 μL of the supernatant and the complete pellet on a 10%–20% Novex Tricine pre-cast gel (Invitrogen, USA). Electrophoresis was performed at 120 V for 90 min. The gel was stained using Coomassie brilliant blue (Invitrogen), and images were obtained using a Gel Doc Imager (Bio-Rad, USA). The experiment was performed three times.

DLS

The hydrodynamic radii of peptide particles in solution were measured using a DynaPro Plate Reader (WYATT Technology, USA) equipped with a temperature-controlled chamber set at 25°C. We analyzed 30 μL of each sample used for the solubility study. Each measurement was performed in triplicate with 10 sub-runs. The hydrodynamic radii were analyzed using Dynamics version 7.19 software. The results were expressed as mean ± SD obtained from three independent experiments.

Hemolysis assay

Erythrocytes were isolated from blood collected from healthy donors in tubes containing lepirudine (50 μg mL^{-1}). Briefly, blood was centrifuged at 250 × *g* for 10 min, plasma was discarded, and RBCs were washed three times in 10 mM Tris at pH 7.4 containing 150 mM NaCl. Cells were diluted 100 times in the same buffer and 100 μL of cell suspension was added to a round-bottom 96-well plate containing 100 μL peptides at the indicated concentration. After 1 h incubation at 37°C and 5% CO_2, the plate was centrifuged, and the absorbance at 450 nm was measured. The percentage of hemolysis was calculated by using formula reported in Petruk et al.[14]

To evaluate the hemolytic effect of the peptides on the whole blood, blood was collected as above. Then, 50 μL blood was transferred to a tube containing 150 μL peptides previously diluted in RPMI-1640-GlutaMAX-I without phenol red (Gibco, Thermo Scientific, USA). Blood diluted in RPMI-1640 (1:4) was used as a negative control, while blood (50 μL) mixed with 150 μL 5% Tween 20 was used as a positive control. After 1 h incubation at 37°C and 5% CO_2, the tubes were centrifuged at 800 × *g*, 100 μL of each sample in duplicate was transferred to a flat-bottom 96-well plate, and the absorbance at 450 nm was measured. The percentage of hemolysis was calculated as in Petruk et al.[14] The data shown are mean ± SEM obtained from four independent experiments all made in triplicate. For each experiment, blood from a different donor was used.

NF-κB activation assay

THP1-XBlue-CD14 reporter cells (InvivoGen, USA) were subcultured in phenol red RPMI media, supplemented with 10% (v/v) heat-inactivated FBS, 1% (v/v) antibiotic-antimycotic solution, Zeocin (200 μg mL^{-1}), and G418 (250 μg mL^{-1}). For the experiment, 180 μL (1 × 10^6 cells mL^{-1}) of cell suspension was seeded in 96-well plates in media without Zeocin and G418. *E. coli* LPS (100 ng mL^{-1}, Sigma-Aldrich, USA) was then added with and without the peptides at the indicated concentration (20 μL total volume). Cells were incubated at 37°C and 5% CO_2 for 20 h. NF-κB activation was determined by mixing 20 μL of supernatant with 180 μL SEAP detection reagent (Quanti-Blue, InvivoGen), as indicated in the manufacturer's instructions (InvivoGen). Then, the absorbance was measured at 600 nm. The data shown are mean ± SD obtained from four independent experiments, all made in triplicate.

Cell viability assay

The cells remaining from the NF-κB assay were used to determine the cell viability. Briefly, 20 μL of MTT (3-(4,5-dimethylthiazol-2-yl)-2,5-diphenyltetrazolium bromide) solution (5 mg mL^{-1}) were added to the cells and incubated for 90 min at 37°C. At the end of incubation, the supernatant in the wells was carefully removed, and the formazan-formed crystals were dissolved in 100 μL dimethyl sulfoxide (Duchefa Biochemie, the Netherlands). The absorbance at 550 nm was then measured. A positive control was obtained by adding a lysis buffer (Thermo Scientific) to the untreated cells and incubating at 37°C for 40 min before adding MTT. The absorbance value for untreated cells was considered to be 100%, and values for other treatments are shown in comparison to the untreated cells. The data shown are mean ± SD obtained from four independent experiments, all made in triplicate.

Whole-blood assay

Blood, collected as for hemolysis assay, was diluted 1:4 with RPMI-1640-GlutaMax I (Gibco, Thermo Scientific) without phenol red. A 1-mL aliquot of blood mixture was distributed in a 24-well plate. Then, 10 μL of each peptide was added to the blood solution to reach the indicated concentrations. The plate was gently shaken, and 10 μL *E. coli* LPS (100 ng mL^{-1} final concentration; Sigma-Aldrich) was added to the wells. The plate was incubated at 37°C for 24 h. After incubation, plasma was collected and stored at −80°C before analysis.

Cytokines assay

Plasma obtained from blood experiment was used to evaluate cytokine release. Human inflammation DuoSet ELISA Kit (R&D Systems, USA) specific for tumor necrosis factor α (TNF-α) and interleukin-1β (IL-1β) was used according to the manufacturer's instructions. Absorbance was measured at a wavelength of 450 nm. The data shown are mean ± SEM obtained from four independent experiments.

Cytokines (TNF-α, interferon-γ, monocyte chemoattractant protein 1, IL-10, and IL-6) in murine plasma were measured by flow cytometry according to the manufacturer's instructions, using the Mouse Inflammation Kit (Becton Dickinson AB, USA).

Mouse model of endotoxin shock

Male C57BL/6 mice (11–12 weeks, 22 ± 5 g) were stimulated intraperitoneally (i.p.) by sublethal dose (6 mg kg^{-1} body weight) *E. coli* 0111:B4 LPS. After 30 min, 10 μg sHVF18 or sK-KVF18 (in 10 mM NaOAc at pH 5) was injected i.p. into the mice. After 20 h, the mice were anesthetized by isoflurane, and the blood was collected by cardiac puncture and stored at −80°C until analysis of cytokines by flow cytometry.

Murine model of polymicrobial sepsis

BALB/c tg(NF-κB-RE-Luc)-Xen reporter mice (Taconic, 10–12 weeks old) or male C57BL/6 mice (Janvier, 8–9 weeks old) were used for the study. Mice were anesthetized with an i.p. injection of ketamine (60 mg kg^{-1}) and xylazine (10 mg kg^{-1}) solution. Hair from the abdomen was shaved with a rodent clipper and disinfected by wiping with a 70% alcohol swab. All procedures were performed under sterile conditions. A small incision (1–1.5 cm) was made along the midline, and the cecum was exposed. Using a 6.0 silk suture, one-third of the cecum was ligated and perforated twice with a 22G needle. A small amount of fecal material was released by gently squeezing the cecum from the perforated sites. The cecum was then returned to the peritoneal cavity. The peritoneum was closed with a 6.0 silk suture. After this, the skin was closed with skin wound clips (AgnThos, Sweden). Resuscitation was achieved by injecting 1 mL prewarmed saline subcutaneously. Mice were then kept on a heating pad for recovery. Immediately after recovery, mice were returned to their respective cages. The mice received 100 μg sHVF18 or sK-KVF18 (in 100 μL water) i.p. every 12 h, over a period of 24 h (for BALB/c tg(NF-κB-RE-Luc)-Xen reporter mice, used for *in vivo* imaging) or 5 days (for C57BL/6 mice, used for survival study). The first dosage was given 30 min after the cecal ligation and puncture (CLP) procedure. The control group was given 100 μL of water i.p. To non-invasively analyze NF-κB activation, we used an In Vivo Imaging System (IVIS Spectrum, PerkinElmer Life Sciences, USA). Fifteen minutes before the IVIS imaging, mice were i.p. given 100 μL of D-luciferin (150 mg kg^{-1} body weight). Bioluminescence from the mice was detected and quantified using Living Image 4.0 software (PerkinElmer Life Sciences).

TI

TI of different peptides was calculated by using data from hemolysis in whole blood and cytokines assays, following Equation 1:

$$TI = \frac{IC_{50}\ \text{hemolysis}}{IC_{50}\ TNF - \alpha} \quad \text{(Equation 1)}$$

where half-maximal inhibitory concentration (IC_{50}) hemolysis is the concentration of peptide able to induce 50% of hemolysis, and IC_{50} TNF-α is the concentration of peptide determining 50% inhibition of TNF-α production in LPS-stimulated blood with respect to control.

MST

Five microliters of LPS-FITC (fluorescein isothiocyanate; 5 μg mL^{-1}, Sigma-Aldrich) was incubated with increasing concentrations of different peptides (0.015–500 μM) in 10 mM Tris at pH 7.4 in a ratio of 1:1. The sample was then loaded into standard glass capillaries (Monolith NT Capillaries, NanoTemper Technologies, Germany), and MST analysis was performed on a NanoTemper Monolith NT.115 apparatus (NanoTemper Technologies). The light-emitting diode and infrared laser were set to 80%.

In another set of experiments, recombinant CD14 (20 μM) was labeled using a Monolith NT Protein labeling kit RED–NHS (NanoTemper Technologies) according to the manufacturer's protocol. Five microliters of 21 nM labeled CD14 was incubated with increasing concentrations of different peptides (0.03–1,000 μM) in 10 mM Tris at pH 7.4 in a ratio of 1:1. The experiment was carried out as described above. The results shown are mean ± SD of four to six measurements.

CG MD simulations of peptide aggregation

The NMR structure of sHVF18[14] was used as the template to model lysine- and arginine-substituted TCPs. The N-terminal histidine residue was replaced with lysine or arginine to generate sKVF18 or sRVF18, respectively. Similarly, sK-KVF18 and sR-RVF18 were built by replacing the histidine with two residues of lysine and arginine, respectively. Modeller 9.21[38] was used to build these models, and the best models were chosen based on having the lowest discrete optimized protein energy (DOPE) score.[39] The Martini 2 forcefield[40] was used to generate CG representations of the peptides, while the secondary and tertiary structures were retained using an elastic network based on the ElNeDYn parameters.[41] The staple was added between residues 10 and 14 for sKVF18 and sRVF18, and between residues 11 and 15 for sK-KVF18 and sR-RVF18, using a similar approach described in our previous study.[14]

To simulate peptide aggregation, 10 copies of each peptide were inserted into a 15 × 15 × 15-nm box with an initial distance of at least 2 nm from one another. Polarizable Martini water particles[42] were added to solvate the box, and 0.15 M NaCl was used to neutralize the system. We next performed steepest descent energy minimization and a 10-ns equilibration simulation with positional restraints (force constant, F_c = 1,000 kJ mol^{-1} nm^{-2}) applied to the backbone particles of the peptides. Three independent 5-μs production simulations with different initial velocities were conducted for each peptide. The temperature of the system was kept at 310 K using a velocity-rescaling thermostat,[43] while the pressure was maintained at 1 atm by an isotropic coupling to a Parrinello-Rahman barostat.[44] The particle mesh Ewald (PME) method was used to calculate electrostatic interactions with a relative dielectric constant of 2.5.[45] The van der Waals interactions were truncated using the potential shift Verlet scheme with a short-range cutoff of 1.1 nm. A 10-fs integration time step was used for these production runs.

CG MD simulations of peptides with lipid A aggregate

To understand the interaction between modified TCPs with LPS, we simulated the stapled peptide with a large lipid A aggregate as described previously.[14] Briefly, 30 copies of the stapled peptides were added to a large 20 × 20 × 20-nm box containing a Ca^{2+}-bound

lipid A aggregate at a minimum distance of 2 nm away from the lipid surface. Martini water particles were added, and an additional 0.15 M NaCl salt was used to neutralize the whole system. Steepest decent energy minimization was then performed. For each peptide, we conducted a 50-ns equilibration simulation, whereby positional restraints were applied on the backbone particles of the peptides and all particles of lipid A, with F_cs of 1,000 kJ mol^{-1} nm^{-2} and 500 kJ mol^{-1} nm^{-2}, respectively. Subsequently, three independent 10-μs production runs were conducted with different initial velocity distributions. The velocity-rescaling thermostat was used to maintain the temperature at 310 K,[43] while an isotropic coupling to a Parrinello-Rahman barostat was used to maintain the pressure at 1 atm.[44] The reaction field method was used to compute electrostatic interactions, while van der Waals interactions were truncated using the potential shift Verlet scheme, both with a 1.1-nm short-range cutoff. An integration time step of 10 fs was used.

All atom molecular dynamics simulations of peptide bound to CD14

In our previous study, we performed microsecond timescale all atom MD simulations of sHVF18 bound to CD14. Clustering analysis was then conducted on the trajectories to obtain a representative structure of the CD14-sHVF18 complex.[14] This representative structure was used as the template to model CD14 bound to the modified TCPs. Similar to our CG models described above, the N-terminal histidine residue was replaced with either one or two lysine or arginine residues using Modeller 9.21, and the best models were selected based on the DOPE score. To generate a model of sVFR17 bound to CD14, the N-terminal histidine residue was deleted using PyMOL. To generate a model of mutant CD14, residues W45, Y82, and F69 were mutated to alanine using CHARMM-GUI. Peptide-bound CD14 was prepared for MD simulations using the CHARMM-GUI protocols[46,47] and the CHARMM36m forcefield.[48] Briefly, the CD14-peptide complex was solvated with TIP3P water, neutralized with 0.15 M NaCl salt, and subjected to energy minimization using the steepest descent method. We then performed a short 125-ps equilibration simulation with positional restraints (F_c = 400 kJ mol^{-1} nm^{-2}) applied to the backbone atoms. Three independent 1-μs production simulations were subsequently conducted with different initial velocities. The Nosé-Hoover thermostat[49,50] was used to maintain the temperature of the system at 310 K, while the pressure was maintained at 1 atm using isotropic coupling to a Parrinello-Rahman barostat.[44] Electrostatic interactions were computed using the PME method.[45] The van der Waals interactions were truncated at 1.2 nm, with a force switch function applied between 1.0 and 1.2 nm. A 2-fs time step was used, with constraints based on the LINCS algorithm applied to all covalent bonds involving hydrogen atoms.[51] All simulations were performed using GROMACS 2021[52] and visualized in VMD.[53]

Evolutionary conservation analysis of thrombin and CD14

The full protein sequence of human prothrombin was obtained from UniProt (UniProt: P00734). We searched for prothrombin protein sequences from other vertebrates in the UniProt database and generated a multiple sequence alignment in Jalview[54] using the ProbCons algorithm.[55] The degree of sequence conservation was mapped to the crystal structure of wild-type human thrombin (PDB: 3U69)[56] using the VMD Multiseq tool.[57] Using the protein-protein BLAST (blastp) algorithm[58] and the sequence of human prothrombin as a query sequence, we searched for sequences of other homologous proteins in humans. The sequences of homologous proteins were aligned using the method described above, and a phylogenetic tree was generated using the PhyML and TreeDyn algorithms.[59] Selected homologous proteins from the same tree branch as prothrombin were analyzed further. We searched for sequences of each homolog in other vertebrates in the UniProt database and generated a multiple sequence alignment to create a consensus sequence for each protein. Using similar methods on CD14 protein sequences, we generated a multiple sequence alignment and a phylogenetic tree for CD14 across different vertebrates based on the human sequence (Uniprot: P08571).

Selection pressure of the F2 gene coding for thrombin was calculated as the ratio of non-synonymous to synonymous substitutions (dN/dS) of the mRNA transcripts. Sequences of human transcripts were obtained from GenBank (GenBank: NM_000506.5) and aligned to transcripts from other vertebrates using the PAL2NAL webserver (https://www.bork.embl.de/pal2nal/).[60] The dN/dS ratios were calculated using the YN00 module in PAMLX software.[61] Finally, per-codon dN/dS ratios along the transcripts were determined using the Fixed Effects Likelihood tool on Datamonkey (https://www.datamonkey.org).[62,63]

Statistical analysis

All *in vitro* assays are biological replicates and were repeated at least three times. Data are presented as mean ± SD or SEM. Differences in the mean between the two groups were analyzed using Student's *t* test. To compare means between more than two groups, a one-way ANOVA with post hoc (Tukey) was used. Statistical analysis, as indicated in each figure legend, was performed using GraphPad Prism software version 10. $p < 0.05$ were considered statistically significant.

Data and code availability

The data supporting the findings of this study are available within the article and in the supplemental information. The original experimental data that support the findings of this work are available from the corresponding author upon request.

ACKNOWLEDGMENTS

The authors thank Ann-Charlotte Strömdahl for excellent technical assistance with the THP-I cells, RBCs, and flow cytometry. Céleste Sele and Wolfgang Knecht from the Lund University Protein Production Platform (LP3) are acknowledged for the production of recombinant CD14. The authors also acknowledge Mats Leeman at SOLVE Research & Consultancy AB (Medicon Village, Lund, Sweden) for excellent support with the DLS measurements. Simulations were performed on resources of the National Supercomputing Center, Singapore (https://www.nscc.sg), and the supercomputer, Fugaku, was provided by RIKEN through the HPCI System Research Project (Project ID: hp240347). This work was supported by grants from the Swedish Research Council (projects 2020-02016, 2021–06388, and 2024–03349), Edvard Welanders Stiftelse and Finsenstiftelsen (Hudfonden), the Royal Physiographic Society, Österlund and Mats

Paulsson Foundations, Stiftelsen Clas Groschinskys minnesfond, Magnus Bergvalls Stiftelse, Vinnova, and the Swedish Government Funds for Clinical Research. We also acknowledge BII (A*STAR) core funds.

AUTHOR CONTRIBUTIONS

G.P. and A.S. conceived the project. G.P., F.S., P.J.B., and A.S. designed the experiments. G.P. performed the experiments involving SDS-PAGE, blood assays, endotoxin shock model, and DLS. M.P. designed and performed the polymicrobial sepsis experiments. J.P. performed the MST assay. F.S. and P.J.B. performed the computational simulations. G.P. and F.S. wrote the manuscript. All the authors discussed the results and revised and commented on the final manuscript.

DECLARATION OF INTERESTS

The authors declare the following competing interests: A.S. is a founder of in2cure AB, a parent company of Xinnate AB, companies that are developing therapies based on thrombin-derived peptides and variants. G.P. is employed part-time (20%) by Xinnate AB. The TCP family, including stapled variants, is patent protected (patent applicant: In2cure AB, Sweden; inventors: G.P. and A.S.). The patent application (WO/2023/067167, PCT/EP2022/079429) was published on April 27, 2023, following an international filing on October 21, 2022. This manuscript covers aspects of the invention, which involves thrombin-derived peptides with at least one internal covalent linkage between the side chains of two non-neighboring internal amino acids, conferring anti-inflammatory properties. The described sequences are included in the patent application.

SUPPLEMENTAL INFORMATION

Supplemental information can be found online at https://doi.org/10.1016/j.ymthe.2025.09.033.

REFERENCES

1. Sharma, K., Sharma, K.K., Sharma, A., and Jain, R. (2023). Peptide-based drug discovery: Current status and recent advances. Drug Discov. Today *28*, 103464. https://doi.org/10.1016/j.drudis.2022.103464.

2. Rossino, G., Marchese, E., Galli, G., Verde, F., Finizio, M., Serra, M., Linciano, P., and Collina, S. (2023). Peptides as Therapeutic Agents: Challenges and Opportunities in the Green Transition Era. Molecules *28*, 7165. https://doi.org/10.3390/molecules28207165.

3. Otvos, L., Jr., and Wade, J.D. (2023). Big peptide drugs in a small molecule world. Front. Chem. *11*, 1302169. https://doi.org/10.3389/fchem.2023.1302169.

4. Migon, D., Neubauer, D., and Kamysz, W. (2018). Hydrocarbon Stapled Antimicrobial Peptides. Protein J. *37*, 2–12. https://doi.org/10.1007/s10930-018-9755-0.

5. Mourtada, R., Herce, H.D., Yin, D.J., Moroco, J.A., Wales, T.E., Engen, J.R., and Walensky, L.D. (2019). Design of stapled antimicrobial peptides that are stable, nontoxic and kill antibiotic-resistant bacteria in mice. Nat. Biotechnol. *37*, 1186–1197. https://doi.org/10.1038/s41587-019-0222-z.

6. Li, Y., Wu, M., Fu, Y., Xue, J., Yuan, F., Qu, T., Rissanou, A.N., Wang, Y., Li, X., and Hu, H. (2024). Therapeutic stapled peptides: Efficacy and molecular targets. Pharmacol. Res. *203*, 107137. https://doi.org/10.1016/j.phrs.2024.107137.

7. Gray, A., and Sharara, F. (2022). Global and regional sepsis and infectious syndrome mortality in 2019: a systematic analysis. Lancet Glob. Health *10*, S2.

8. Papareddy, P., Rydengård, V., Pasupuleti, M., Walse, B., Mörgelin, M., Chalupka, A., Malmsten, M., and Schmidtchen, A. (2010). Proteolysis of human thrombin generates novel host defense peptides. PLoS Pathog. *6*, e1000857. https://doi.org/10.1371/journal.ppat.1000857.

9. Saravanan, R., Holdbrook, D.A., Petrlova, J., Singh, S., Berglund, N.A., Choong, Y. K., Kjellström, S., Bond, P.J., Malmsten, M., and Schmidtchen, A. (2018). Structural basis for endotoxin neutralisation and anti-inflammatory activity of thrombin-derived C-terminal peptides. Nat. Commun. *9*, 2762. https://doi.org/10.1038/s41467-018-05242-0.

10. Puthia, M., Butrym, M., Petrlova, J., Strömdahl, A.C., Andersson, M.Å., Kjellström, S., and Schmidtchen, A. (2020). A dual-action peptide-containing hydrogel targets wound infection and inflammation. Sci. Transl. Med. *12*, eaax6601. https://doi.org/10.1126/scitranslmed.aax6601.

11. Puthia, M., Petrlova, J., Petruk, G., Butrym, M., Samsudin, F., Andersson, M.Å., Strömdahl, A.C., Wasserstrom, S., Hartman, E., Kjellström, S., et al. (2023). Bioactive Suture with Added Innate Defense Functionality for the Reduction of Bacterial Infection and Inflammation. Adv. Healthc. Mater. *12*, e2300987. https://doi.org/10.1002/adhm.202300987.

12. Stromdahl, A.C., Ignatowicz, L., Petruk, G., Butrym, M., Wasserstrom, S., Schmidtchen, A., and Puthia, M. (2021). Peptide-coated polyurethane material reduces wound infection and inflammation. Acta Biomater. *128*, 314–331. https://doi.org/10.1016/j.actbio.2021.04.045.

13. Lundgren, S., Wallblom, K., Fisher, J., Erdmann, S., Schmidtchen, A., and Saleh, K. (2023). Study protocol for a phase 1, randomised, double-blind, placebo-controlled study to investigate the safety, tolerability and pharmacokinetics of ascending topical doses of TCP-25 applied to epidermal suction blister wounds in healthy male and female volunteers. BMJ Open *13*, e064866. https://doi.org/10.1136/bmjopen-2022-064866.

14. Petruk, G., Puthia, M., Samsudin, F., Petrlova, J., Olm, F., Mittendorfer, M., Hyllén, S., Edström, D., Strömdahl, A.C., Diehl, C., et al. (2023). Targeting Toll-like receptor-driven systemic inflammation by engineering an innate structural fold into drugs. Nat. Commun. *14*, 6097. https://doi.org/10.1038/s41467-023-41702-y.

15. Petruk, G., Petrlova, J., Samsudin, F., Giudice, R.D., Bond, P.J., and Schmidtchen, A. (2020). Concentration- and pH-Dependent Oligomerization of the Thrombin-Derived C-Terminal Peptide TCP-25. Biomolecules *10*, 1572. https://doi.org/10.3390/biom10111572.

16. Yang, C.H., Chen, Y.C., Peng, S.Y., Tsai, A.P.Y., Lee, T.J.F., Yen, J.H., and Liou, J.W. (2018). An engineered arginine-rich alpha-helical antimicrobial peptide exhibits broad-spectrum bactericidal activity against pathogenic bacteria and reduces bacterial infections in mice. Sci. Rep. *8*, 14602. https://doi.org/10.1038/s41598-018-32981-3.

17. Gallivan, J.P., and Dougherty, D.A. (1999). Cation-pi interactions in structural biology. Proc. Natl. Acad. Sci. USA *96*, 9459–9464. https://doi.org/10.1073/pnas.96.17.9459.

18. Liao, S.M., Du, Q.S., Meng, J.Z., Pang, Z.W., and Huang, R.B. (2013). The multiple roles of histidine in protein interactions. Chem. Cent. J. *7*, 44. https://doi.org/10.1186/1752-153X-7-44.

19. Holdbrook, D.A., Singh, S., Choong, Y.K., Petrlova, J., Malmsten, M., Bond, P.J., Verma, N.K., Schmidtchen, A., and Saravanan, R. (2018). Influence of pH on the activity of thrombin-derived antimicrobial peptides. Biochim. Biophys. Acta Biomembr. *1860*, 2374–2384. https://doi.org/10.1016/j.bbamem.2018.06.002.

20. Holliday, G.L., Mitchell, J.B.O., and Thornton, J.M. (2009). Understanding the functional roles of amino acid residues in enzyme catalysis. J. Mol. Biol. *390*, 560–577. https://doi.org/10.1016/j.jmb.2009.05.015.

21. Perona, J.J., and Craik, C.S. (1995). Structural basis of substrate specificity in the serine proteases. Protein Sci. *4*, 337–360. https://doi.org/10.1002/pro.5560040301.

22. Meng, Z.H., Wolberg, A.S., Monroe, D.M., and Hoffman, M. (2003). The effect of temperature and pH on the activity of factor VIIa: implications for the efficacy of high-dose factor VIIa in hypothermic and acidotic patients. J. Trauma *55*, 886–891. https://doi.org/10.1097/01.TA.0000066184.20808.A5.

23. Al-Amer, O.M. (2022). The role of thrombin in haemostasis. Blood Coagul. Fibrinolysis *33*, 145–148. https://doi.org/10.1097/MBC.0000000000001130.

24. Foley, J.H., and Conway, E.M. (2016). Cross Talk Pathways Between Coagulation and Inflammation. Circ. Res. *118*, 1392–1408. https://doi.org/10.1161/CIRCRESAHA.116.306853.

25. Mansory, E.M., Bhai, P., Stuart, A., Laudenbach, L., Sadikovic, B., and Lazo-Langner, A. (2021). A case of congenital prothrombin deficiency with two concurrent mutations in the prothrombin gene. Res. Pract. Thromb. Haemost. *5*, e12510. https://doi.org/10.1002/rth2.12510.

26. Akhavan, S., Luciani, M., Lavoretano, S., and Mannucci, P.M. (2003). Phenotypic and genetic analysis of a compound heterozygote for dys- and hypoprothrombinaemia. Br. J. Haematol. *120*, 142–144. https://doi.org/10.1046/j.1365-2141.2003.03986.x.

27. van der Plas, M.J.A., Bhongir, R.K.V., Kjellström, S., Siller, H., Kasetty, G., Mörgelin, M., and Schmidtchen, A. (2016). Pseudomonas aeruginosa elastase cleaves a

C-terminal peptide from human thrombin that inhibits host inflammatory responses. Nat. Commun. *7*, 11567. https://doi.org/10.1038/ncomms11567.

28. Dahlman, A., Puthia, M., Petrlova, J., Schmidtchen, A., and Petruk, G. (2021). Thrombin-Derived C-Terminal Peptide Reduces Candida-Induced Inflammation and Infection In Vitro and In Vivo. Antimicrob. Agents Chemother. *65*, e0103221. https://doi.org/10.1128/AAC.01032-21.

29. Hansen, F.C., Kalle-Brune, M., van der Plas, M.J.A., Strömdahl, A.C., Malmsten, M., Mörgelin, M., and Schmidtchen, A. (2015). The Thrombin-Derived Host Defense Peptide GKY25 Inhibits Endotoxin-Induced Responses through Interactions with Lipopolysaccharide and Macrophages/Monocytes. J. Immunol. *194*, 5397–5406. https://doi.org/10.4049/jimmunol.1403009.

30. Hansen, F.C., Strömdahl, A.C., Mörgelin, M., Schmidtchen, A., and van der Plas, M.J. A. (2017). Thrombin-Derived Host-Defense Peptides Modulate Monocyte/Macrophage Inflammatory Responses to Gram-Negative Bacteria. Front. Immunol. *8*, 843. https://doi.org/10.3389/fimmu.2017.00843.

31. Kalle, M., Papareddy, P., Kasetty, G., Mörgelin, M., van der Plas, M.J.A., Rydengård, V., Malmsten, M., Albiger, B., and Schmidtchen, A. (2012). Host defense peptides of thrombin modulate inflammation and coagulation in endotoxin-mediated shock and Pseudomonas aeruginosa sepsis. PLoS One *7*, e51313. https://doi.org/10.1371/journal.pone.0051313.

32. Lim, C.H., Puthia, M., Butrym, M., Tay, H.M., Lee, M.Z.Y., Hou, H.W., and Schmidtchen, A. (2017). Thrombin-derived host defence peptide modulates neutrophil rolling and migration in vitro and functional response in vivo. Sci. Rep. *7*, 11201. https://doi.org/10.1038/s41598-017-11464-x.

33. Petrlova, J., Hansen, F.C., van der Plas, M.J.A., Huber, R.G., Mörgelin, M., Malmsten, M., Bond, P.J., and Schmidtchen, A. (2017). Aggregation of thrombin-derived C-terminal fragments as a previously undisclosed host defense mechanism. Proc. Natl. Acad. Sci. USA *114*, E4213–E4222. https://doi.org/10.1073/pnas.1619609114.

34. Petrlova, J., Petruk, G., Huber, R.G., McBurnie, E.W., van der Plas, M.J.A., Bond, P. J., Puthia, M., and Schmidtchen, A. (2020). Thrombin-derived C-terminal fragments aggregate and scavenge bacteria and their proinflammatory products. J. Biol. Chem. *295*, 3417–3430. https://doi.org/10.1074/jbc.RA120.012741.

35. Vit, O., and Petrak, J. (2017). Integral membrane proteins in proteomics. How to break open the black box? J. Proteomics *153*, 8–20. https://doi.org/10.1016/j.jprot.2016.08.006.

36. Petruk, G., Puthia, M., Petrlova, J., Samsudin, F., Strömdahl, A.C., Cerps, S., Uller, L., Kjellström, S., Bond, P.J., and Schmidtchen, A.A. (2020). SARS-CoV-2 Spike protein binds to bacterial lipopolysaccharide and boosts proinflammatory activity. J. Mol. Cell Biol. *12*, 916–932. https://doi.org/10.1093/jmcb/mjaa067.

37. Morrisett, J.D., David, J.S., Pownall, H.J., and Gotto, A.M., Jr. (1973). Interaction of an apolipoprotein (apoLP-alanine) with phosphatidylcholine. Biochemistry *12*, 1290–1299. https://doi.org/10.1021/bi00731a008.

38. Sali, A., and Blundell, T.L. (1993). Comparative protein modelling by satisfaction of spatial restraints. J. Mol. Biol. *234*, 779–815. https://doi.org/10.1006/jmbi.1993.1626.

39. Shen, M.Y., and Sali, A. (2006). Statistical potential for assessment and prediction of protein structures. Protein Sci. *15*, 2507–2524. https://doi.org/10.1110/ps.062416606.

40. Monticelli, L., Kandasamy, S.K., Periole, X., Larson, R.G., Tieleman, D.P., and Marrink, S.J. (2008). The MARTINI Coarse-Grained Force Field: Extension to Proteins. J. Chem. Theor. Comput. *4*, 819–834. https://doi.org/10.1021/ct700324x.

41. Periole, X., Cavalli, M., Marrink, S.J., and Ceruso, M.A. (2009). Combining an Elastic Network With a Coarse-Grained Molecular Force Field: Structure, Dynamics, and Intermolecular Recognition. J. Chem. Theor. Comput. *5*, 2531–2543. https://doi.org/10.1021/ct9002114.

42. Yesylevskyy, S.O., Schäfer, L.V., Sengupta, D., and Marrink, S.J. (2010). Polarizable water model for the coarse-grained MARTINI force field. Plos Comput. Biol. *6*, e1000810. https://doi.org/10.1371/journal.pcbi.1000810.

43. Bussi, G., Donadio, D., and Parrinello, M. (2007). Canonical sampling through velocity rescaling. J. Chem. Phys. *126*, 014101. https://doi.org/10.1063/1.2408420.

44. Parrinello, M., and Rahman, A. (1981). Polymorphic Transitions in Single Crystals: A New Molecular Dynamics Method. J. Appl. Phys. *52*, 7182–7190. https://doi.org/10.1063/1.328693.

45. Essmann, U., Perera, L., Berkowitz, M.L., Darden, T., Lee, H., and Pedersen, L.G. (1995). A smooth particle mesh Ewald method. J. Chem. Phys. *103*, 8577–8593.

46. Jo, S., Kim, T., Iyer, V.G., and Im, W. (2008). CHARMM-GUI: a web-based graphical user interface for CHARMM. J. Comput. Chem. *29*, 1859–1865.

47. Lee, J., Cheng, X., Swails, J.M., Yeom, M.S., Eastman, P.K., Lemkul, J.A., Wei, S., Buckner, J., Jeong, J.C., Qi, Y., et al. (2016). CHARMM-GUI Input Generator for NAMD, GROMACS, AMBER, OpenMM, and CHARMM/OpenMM Simulations Using the CHARMM36 Additive Force Field. J. Chem. Theor. Comput. *12*, 405–413. https://doi.org/10.1021/acs.jctc.5b00935.

48. Huang, J., and MacKerell, A.D., Jr. (2013). CHARMM36 all-atom additive protein force field: Validation based on comparison to NMR data. J. Comput. Chem. *34*, 2135–2145.

49. Nosé, S. (1984). A unified formulation of the constant temperature molecular dynamics methods. J. Phys. Chem. *81*, 511–519.

50. Hoover, W.G. (1985). Canonical dynamics: Equilibrium phase-space distributions. Phys. Rev. A *31*, 1695–1697.

51. Hess, B., Bekker, H., Berendsen, H.J.C., and Fraaije, J.G.E.M. (1997). LINCS: A linear constraint solver for molecular simulations. J. Comput. Chem. *18*, 1463–1472. https://doi.org/10.1002/(SICI)1096-987X(199709)18:12<1463::AID-JCC4>3.0.CO;2-H.

52. Abraham, M.J., Murtola, T., Páll, S., Páll, S., Smith, J.C., Hess, B., and Lindahl, E. (2015). GROMACS: High performance molecular simulations through multi-level parallelism from laptops to supercomputers. SoftwareX *1–2*, 19–25. https://doi.org/10.1016/j.softx.2015.06.001.

53. Humphrey, W., Dalke, A., and Schulten, K. (1996). VMD: visual molecular dynamics. J. Mol. Graph. *14*, 33.

54. Waterhouse, A.M., Procter, J.B., Martin, D.M.A., Clamp, M., and Barton, G.J. (2009). Jalview Version 2--a multiple sequence alignment editor and analysis workbench. Bioinformatics *25*, 1189–1191. https://doi.org/10.1093/bioinformatics/btp033.

55. Do, C.B., Mahabhashyam, M.S.P., Brudno, M., and Batzoglou, S. (2005). ProbCons: Probabilistic consistency-based multiple sequence alignment. Genome Res. *15*, 330–340. https://doi.org/10.1101/gr.2821705.

56. Figueiredo, A.C., Clement, C.C., Zakia, S., Gingold, J., Philipp, M., and Pereira, P.J.B. (2012). Rational design and characterization of D-Phe-Pro-D-Arg-derived direct thrombin inhibitors. PLoS One *7*, e34354. https://doi.org/10.1371/journal.pone.0034354.

57. Roberts, E., Eargle, J., Wright, D., and Luthey-Schulten, Z. (2006). MultiSeq: unifying sequence and structure data for evolutionary analysis. BMC Bioinformatics *7*, 382. https://doi.org/10.1186/1471-2105-7-382.

58. Altschul, S.F., Gish, W., Miller, W., Myers, E.W., and Lipman, D.J. (1990). Basic local alignment search tool. J. Mol. Biol. *215*, 403–410. https://doi.org/10.1016/S0022-2836(05)80360-2.

59. Dereeper, A., Guignon, V., Blanc, G., Audic, S., Buffet, S., Chevenet, F., Dufayard, J. F., Guindon, S., Lefort, V., Lescot, M., et al. (2008). Phylogeny.fr: robust phylogenetic analysis for the non-specialist. Nucleic Acids Res. *36*, W465–W469. https://doi.org/10.1093/nar/gkn180.

60. Suyama, M., Torrents, D., and Bork, P. (2006). PAL2NAL: robust conversion of protein sequence alignments into the corresponding codon alignments. Nucleic Acids Res. *34*, W609–W612. https://doi.org/10.1093/nar/gkl315.

61. Yang, Z. (2007). PAML 4: phylogenetic analysis by maximum likelihood. Mol. Biol. Evol. *24*, 1586–1591. https://doi.org/10.1093/molbev/msm088.

62. Kosakovsky Pond, S.L., and Frost, S.D.W. (2005). Not so different after all: a comparison of methods for detecting amino acid sites under selection. Mol. Biol. Evol. *22*, 1208–1222.

63. Weaver, S., Shank, S.D., Spielman, S.J., Li, M., Muse, S.V., and Kosakovsky Pond, S.L. (2018). Datamonkey 2.0: A Modern Web Application for Characterizing Selective and Other Evolutionary Processes. Mol. Biol. Evol. *35*, 773–777. https://doi.org/10.1093/molbev/msx335.

YMTHE, Volume *34*

Supplemental Information

The conserved N-terminal histidine in an engineered peptide mediates sepsis treatment efficacy via dual binding to CD14 and LPS

Ganna Petruk, Firdaus Samsudin, Manoj Puthia, Jitka Petrlova, Peter J. Bond, and Artur Schmidtchen

Supplemental Figures

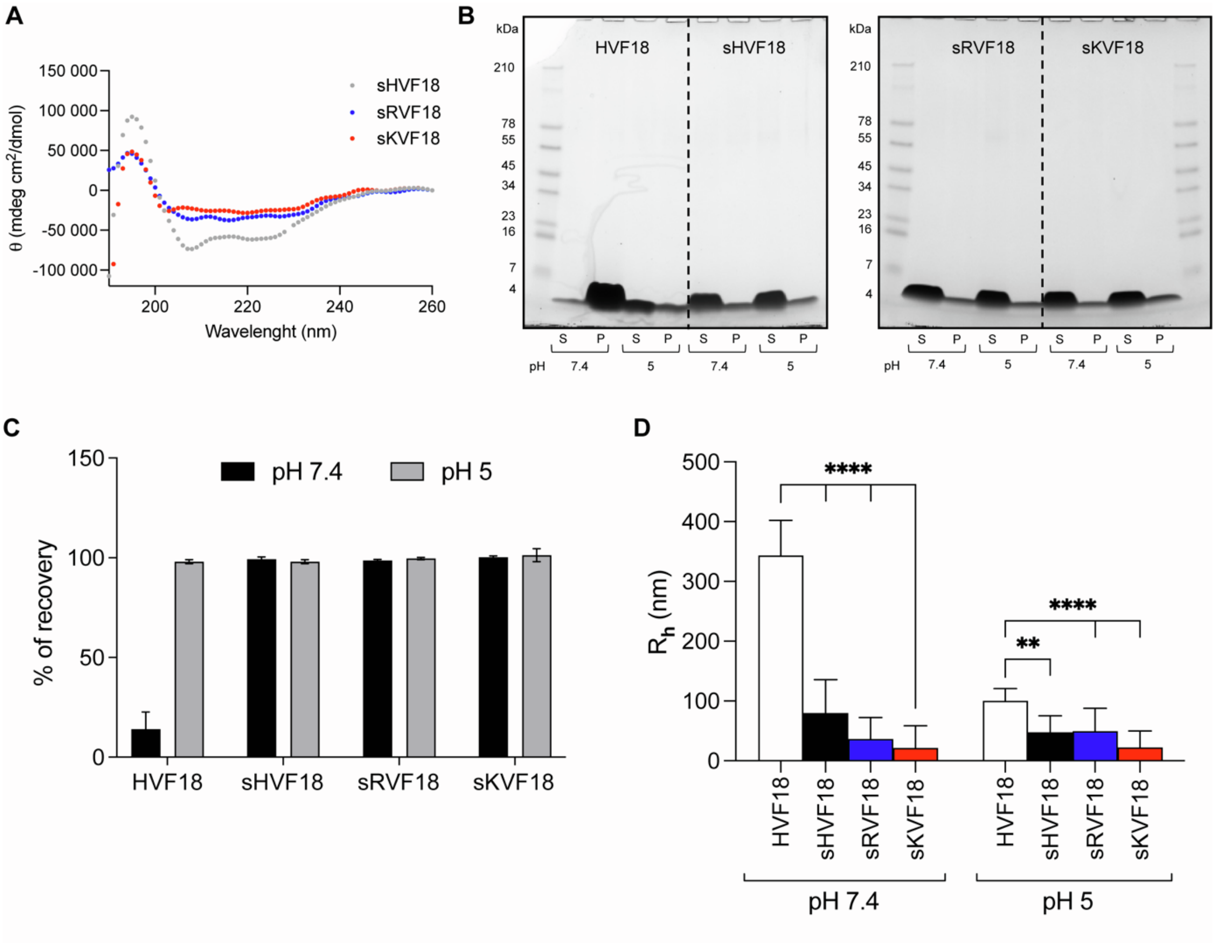

Figure S1 | Solubility of sHVF18 and its variants. (A) Circular dichroism spectra of sHVF18, sRVF18 and sKVF18 dissolved in 10 mM NaOAc at pH 5.0. Data are shown as mean ± SD of 3 different experiments (n = 3). (B) SDS-PAGE of supernatants (S) and pellets (P) from the centrifugation of 30 µL (1 mM) sHVF18 and its variants dissolved in 10 mM Tris at pH 7.4 or 10 mM NaOAc pH 5.0. (C) The graph shows the percentage of peptide recovery ± SD in the supernatant after centrifugation with respect to the starting concentration. (D) Peptides were dissolved in 10 mM Tris at pH 7.4 or 10 mM NaOAc pH 5.0 at 1 mM as final concentration, then hydrodynamic radii of the particles in solution were measured by DLS. The data are presented as mean ± SD (n = 3).

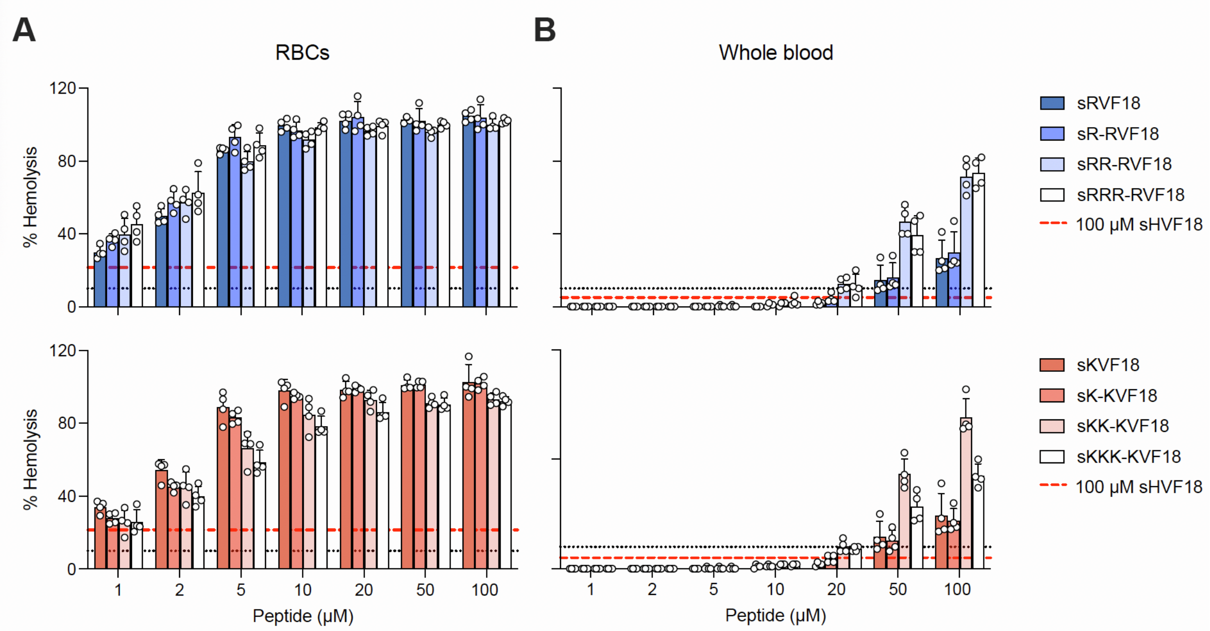

Figure S2 | Hemolytic effect of different sHVF18 variants. (A) The curves show the hemolytic activity of the peptides on erythrocytes (RBCs, A) or whole blood (B). Results are presented as means ± SD of four different experiments (n = 4). For each experiment, blood from a different donor was used. Red dashed line shows the hemolysis induced by 100 µM sHVF18. Black dotted line shows 10% of hemolysis.

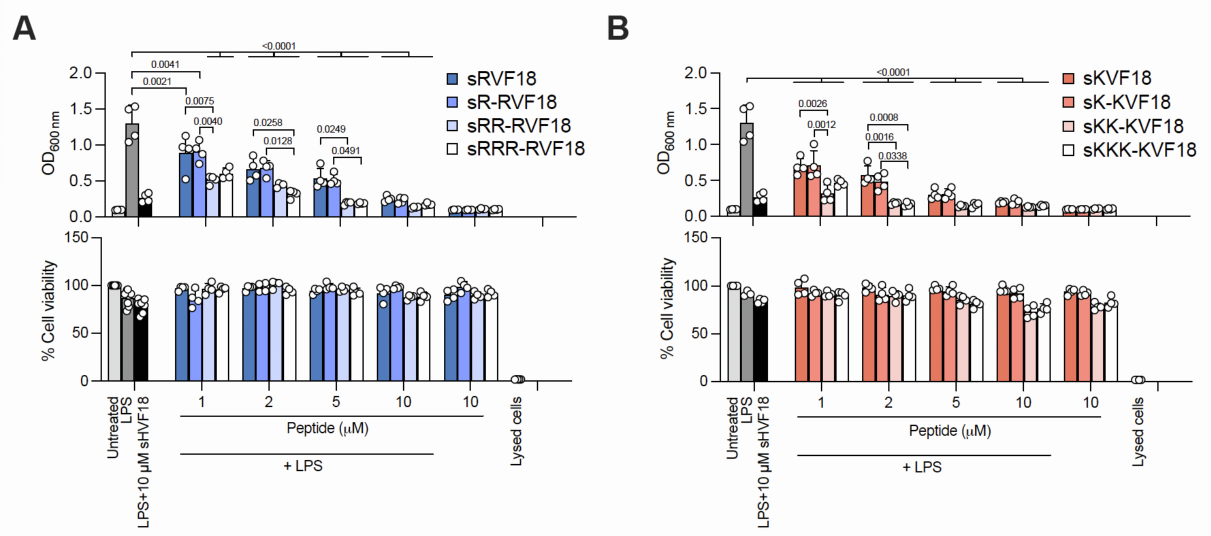

Figure S3 | Anti-inflammatory activity of different sHVF18 variants in THP1-XBlue-CD14 reporter cells. NF-κB activation and cell viability using monocytes stimulated with 100 ng mL^{-1} *E. coli* LPS in the presence or absence of increasing concentrations of arginine (A) or lysin (B) variants of sHVF18, 20 h poststimulation. Results are presented as means ± SD of four experiments (n = 4). P values were determined using an ordinary two-way ANOVA followed by Tukey's multiple comparisons tests using GraphPad Prism software.

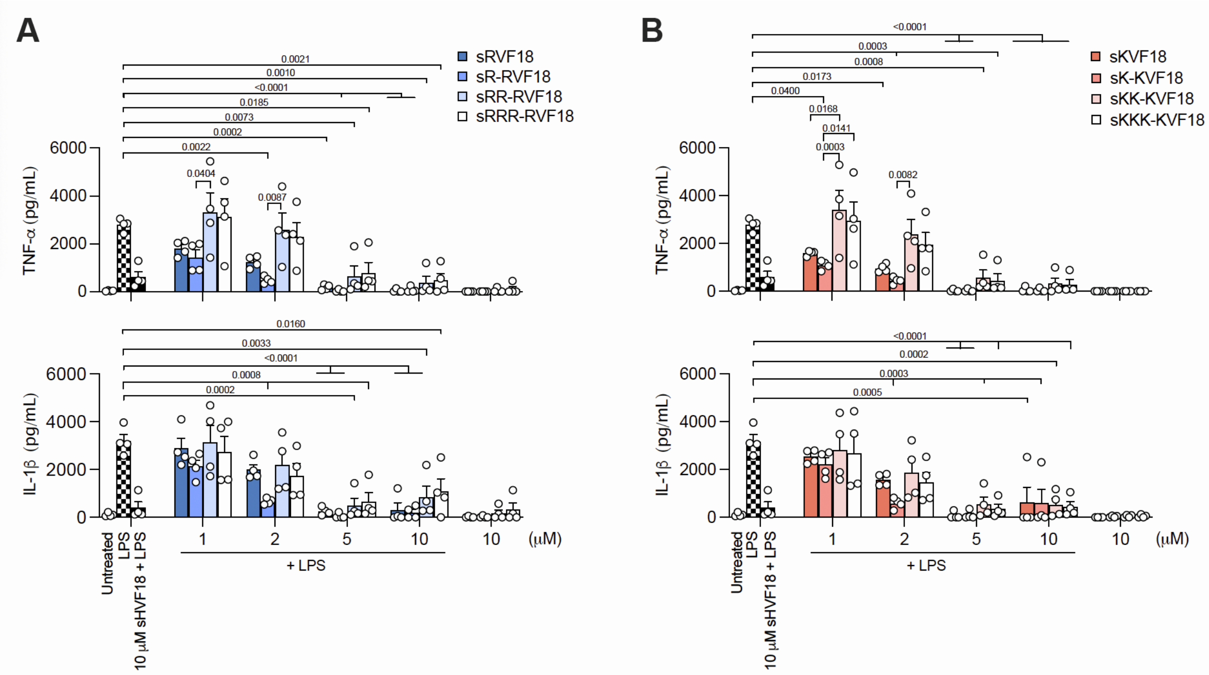

Figure S4 | Anti-inflammatory activity of different sHVF18 variants in human blood. TNF-α and IL-1β release from blood stimulated with 100 ng mL^{-1} *E. coli* LPS in the presence or absence of increasing concentrations of arginine (A) or lysin (B) variants of sHVF18, 24 h poststimulation. Results are presented as means ± SEM of four experiments, each performed with the blood from a different donor (n = 4). P values were determined using an ordinary two-way ANOVA followed by Tukey's multiple comparisons tests using GraphPad Prism software.

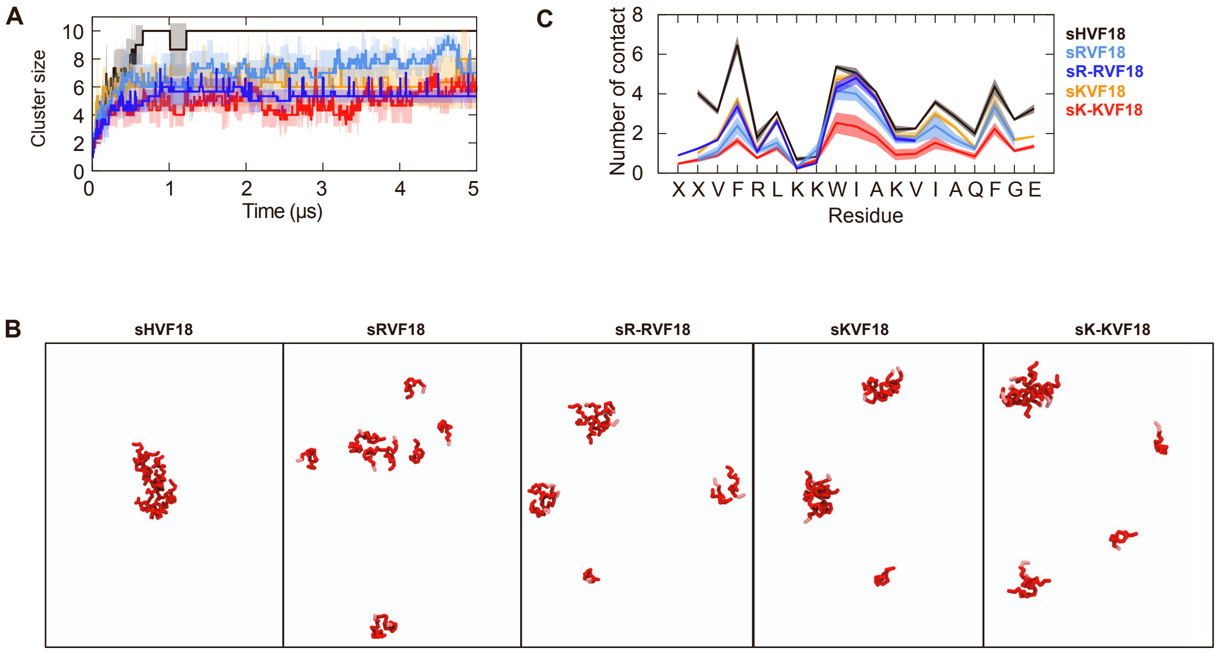

Figure S5 | sHVF18 variants with positively charged N-terminus aggregate less in solution. (A) CG simulations of 10 copies of sHVF18 or N-terminal variant peptides in a box of water were performed. The graph shows comparison of the number of peptides forming the largest cluster throughout the simulations, whereby a cluster size of 10 indicates that all peptides form one cluster. Average values from three independent repeats are shown as thick lines, whereas the standard deviations are depicted as shaded areas. Cut-off distance for cluster size calculation is 0.6 nm. (B) The final snapshots from one of the simulations for each peptide. Peptides are coloured in red with the N-terminal arginine and lysine highlighted in pink. (C) Average number of contacts made by each residue of one peptide with all the other peptides in the system in the last 3 μs of the simulations. Cut-off distance for contact calculation is 0.6 nm.

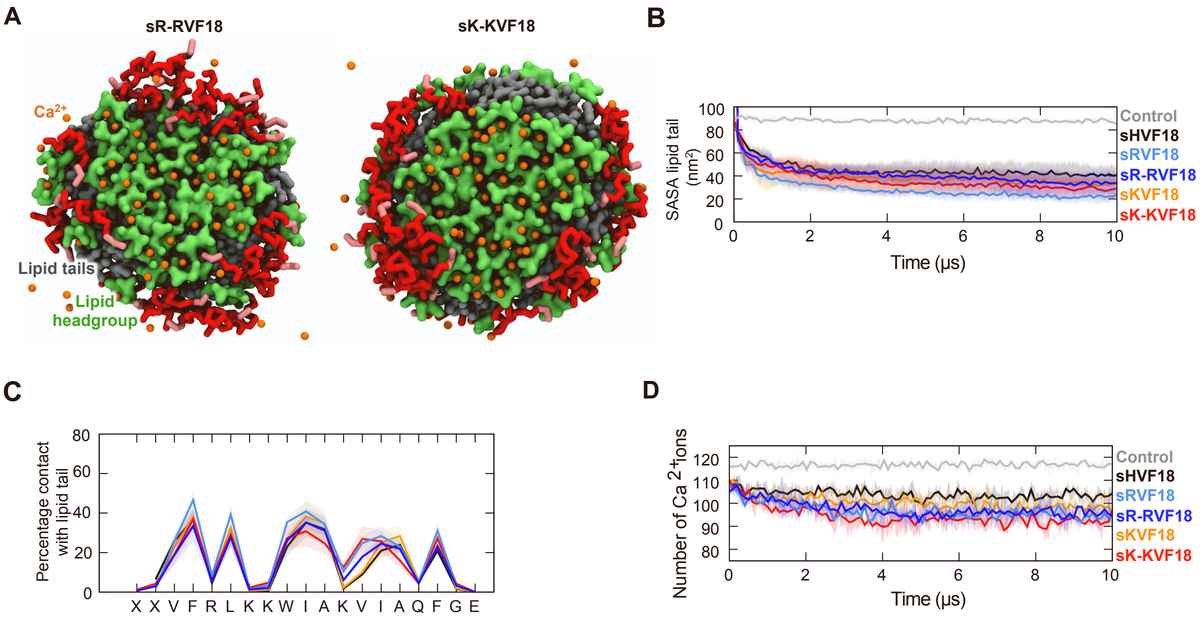

Figure S6 | CG simulations of sHVF18 and N-terminal variants with lipid A aggregate. (A) The final snapshots from one CG simulations of lipid A aggregate with sR-RVF18 and sK-KVF18. Peptides are coloured in red with the N-terminal arginine or lysine residues highlighted in pink. The lipids are shown in surface representation with the headgroup in green and tails in grey. Ca^{2+} ions are shown as orange spheres. (B) Solvent accessible surface area (SASA) of lipid tails throughout the simulations with peptides and without peptides (grey). Thick lines show average over three independent simulations and the standard deviation is depicted as the shaded areas. A probe radius of 0.26 nm was used for the calculation to represent the radius of water particles in the Martini force field. (C) Percentage of contact made by each residue of the peptide with lipid tails. Cut off distance for contact measurement is 0.6 nm. (D) The number of Ca^{2+} ions found within 0.6 nm of the phosphate groups of the lipids.

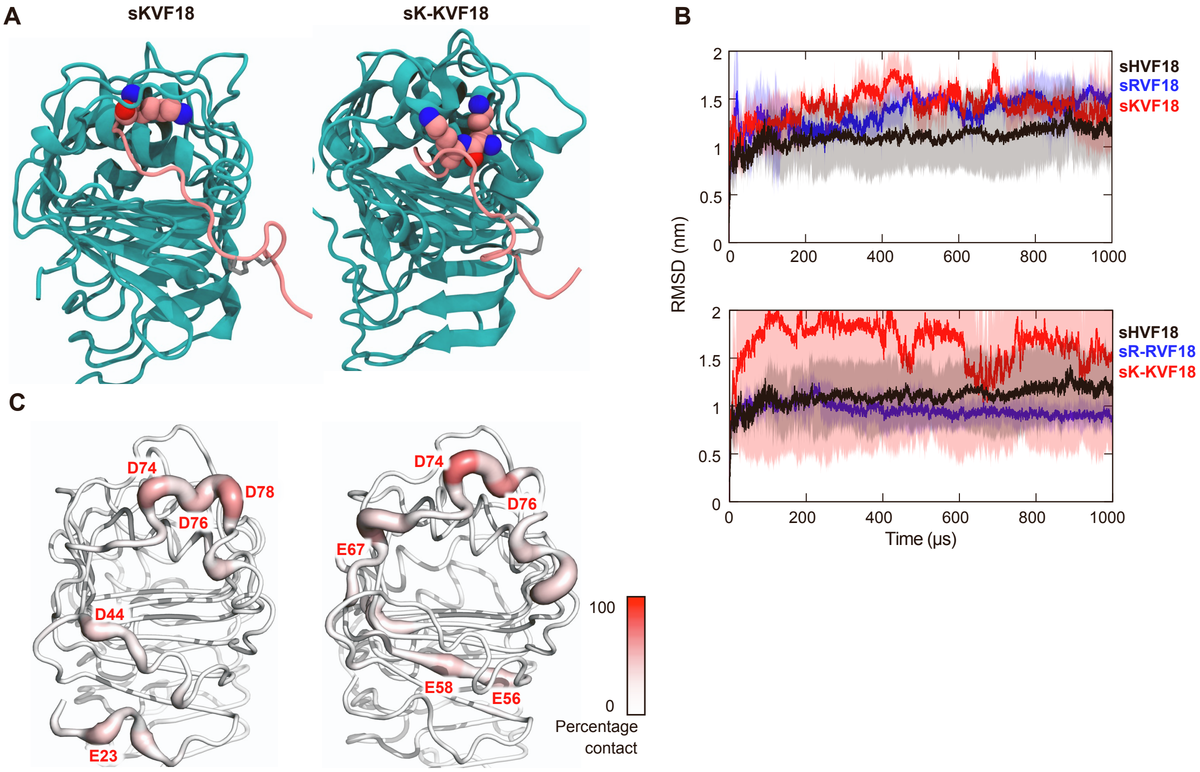

Figure S7 | Simulations of modified peptides bound to CD14. (A) The N-terminal histidine residue was mutated to either one or two lysine residues and three independent 1 μs simulations were performed. The figures show CD14 (cyan) and peptide (pink) with the N-terminal lysine residue(s) depicted in van der Waals representation, taken from the central structure of the top cluster from a clustering analysis performed on concatenated trajectories. (B) RMSD of backbone atoms of the whole peptide after least square fitting to the initial structure of the CD14-peptide complex, compared to the RMSD of sHVF18 (black). Thick lines show average values from three simulations, and the standard deviations are shown as shaded regions. (C) Average percentage of contacts made by CD14 residues with the ε-ammonium group of lysine at the N-terminus of modified peptides from three simulations. The top five residues that made the most contacts are labelled.

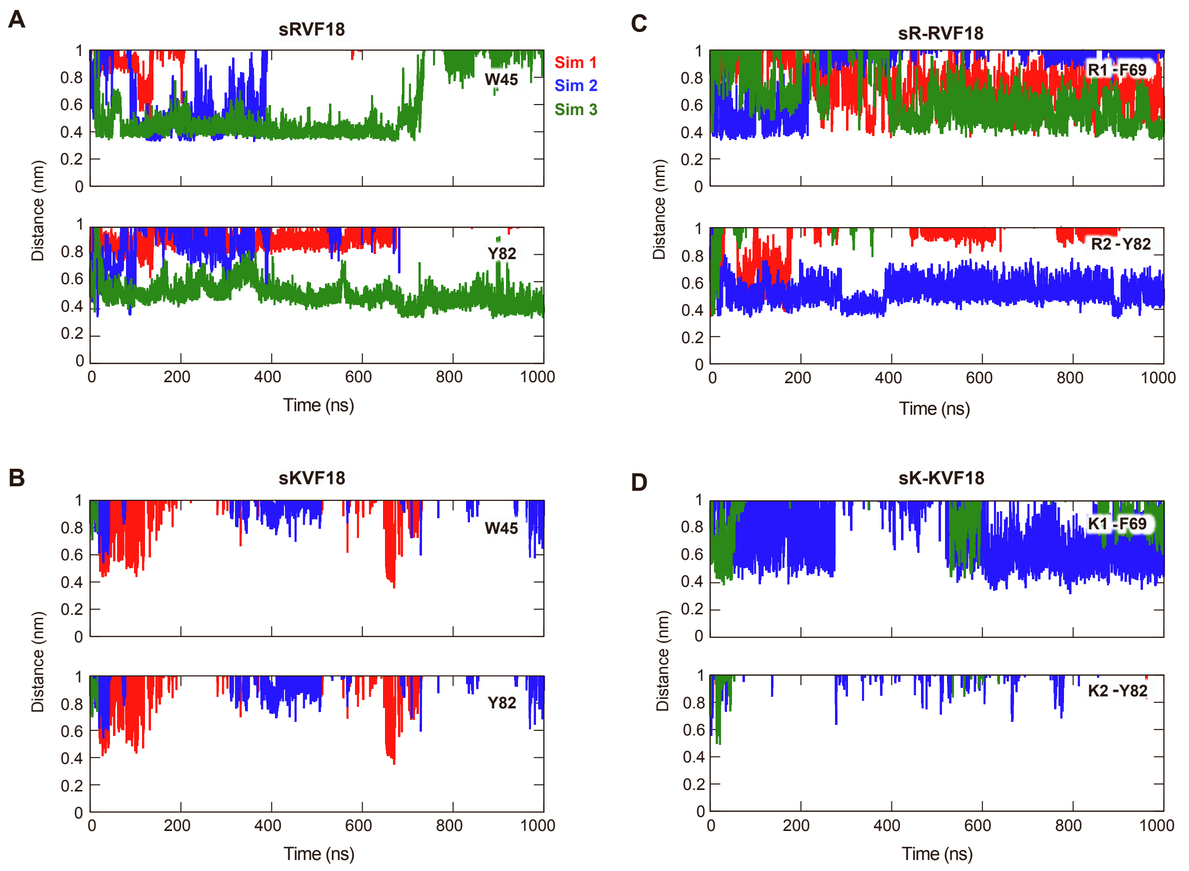

Figure S8 | Distance measurement between N-terminal arginine and lysine residues and aromatic residues on CD14. (A) The distance from the guanidinium group of residue R1 on sRVF18 with the side chain of residues W45 and Y82 on CD14 from three independent simulations. (B) Similar distance measurement performed for the ε-ammonium group of K1 residue on sKVF18. (C) The distance from the guanidinium group of residue R1 (top) and R2 (bottom) on sR-RVF18 with the side chain of residues F69 and Y82, respectively, on CD14 from three independent simulations. (D) Similar distance measurement performed for the ε-ammonium group of N-terminal lysine residues on sK-KVF18.

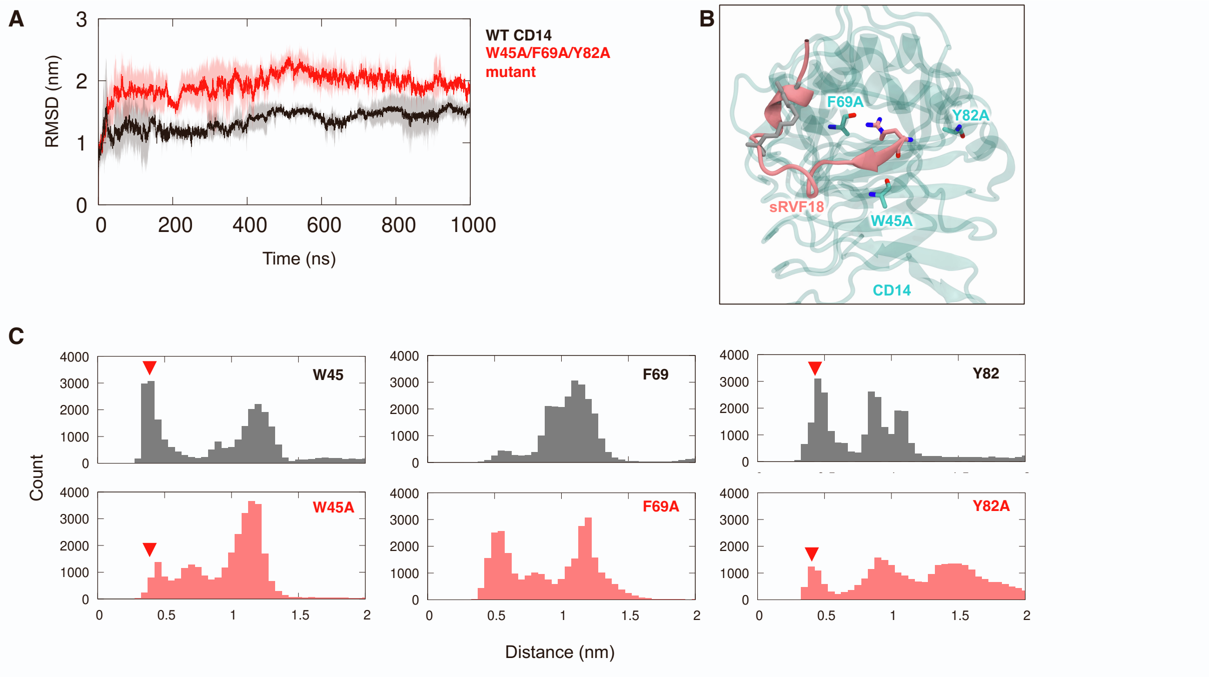

Figure S9 | Simulation of sRVF18 with CD14 mutant. (A) Residues W45, F69, and Y82 on CD14 were mutated to alanine and all-atom MD simulations of CD14 mutant complexed with sRVF18 were performed. The figure shows average RMSDs of backbone atoms of the peptide, comparing simulations with wild-type CD14 (black) and the mutant (red). The RMSDs are calculated after least square fitting to the initial structure of the CD14-peptide complex. The values are averaged from three independent simulations, while the standard deviation is shown as shaded areas. (B) Cluster analysis was performed on concatenated trajectories using an RMSD cut-off of 0.4 nm. The figure shows the central structure of the top cluster with CD14 in cyan and the peptide in pink. The N-terminal arginine as well as mutated residues on CD14 are highlighted in stick representation. (C) The distribution of distance between the side chain of N-terminal arginine on sRVF18 and the side chain of W45, F69, and Y82 from wild-type simulations (black) compared to the alanine mutant (red). Red triangles highlight the loss of interaction between the arginine side chain and residues W45 and Y82 in mutant simulations. The distance values are cumulative from three independent simulations.

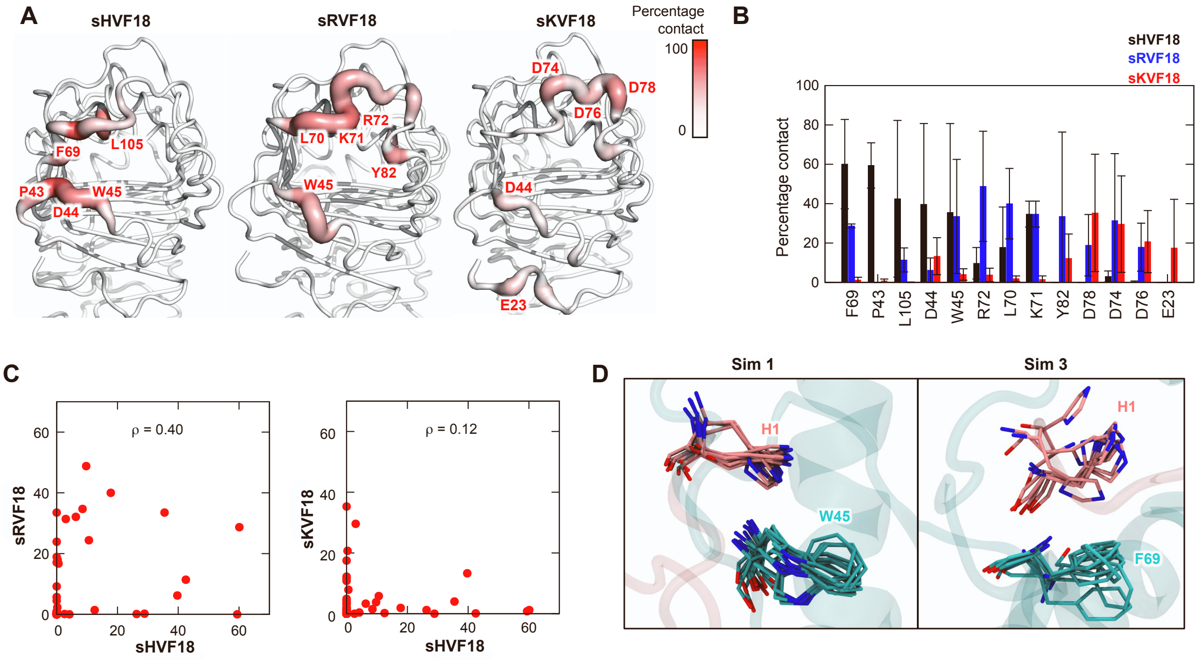

Figure S10 | Comparison of contacts made by sHVF18, sRVF18 and sKVF18 peptides on CD14. (A) Average percentage of contacts made by each residue on CD14 to the imidazole group of histidine, the guanidinium group of arginine, and the ε-ammonium group of lysine at the N-terminus of the respective peptides from three independent 1 μs simulations. The top five residues that made the most contacts are labelled. (B) The values of percentage of contacts made with the top five residues. Error bars show standard deviation between three repeats. (C) Correlation plot of average percentage contacts made by N-terminal residues of sHVF18 with either sRVF18 (left) or sKVF18 (right). The Pearson correlation coefficient values are shown. (D) Overlaid snapshots from sHVF18-CD14 simulations taken every 50 ns from parts of the simulations showing staggered π-π stacking interaction between the N-terminal histidine residue on the peptide (pink) and aromatic residues on CD14 (cyan).

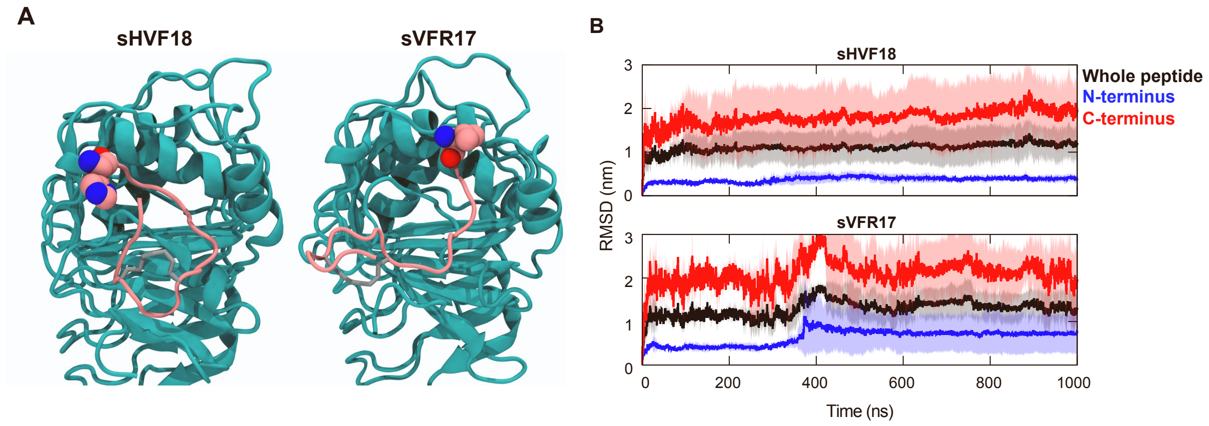

Figure S11 | All-atom MD simulations of CD14 bound to sHVF18 and sVFR17. (A) The figure shows a snapshot of CD14 (cyan) and peptide (pink) with the N-terminal H1 or V1 residue shown in van der Waals representation. The snapshot is taken from the central structure of the top cluster from a clustering analysis performed on concatenated trajectories. (B) RMSD profiles of the modelled sVFR17 compared to sHVF18. The figure shows the RMSD of backbone atoms of the first five residues at the N-terminus (blue), the last five residues at the C-terminus (red), and all residues (black) after least-squares fitting to the backbone atoms of the CD14-peptide complex. Thick lines show average values from three simulations, and the standard deviations are shown as shaded regions.

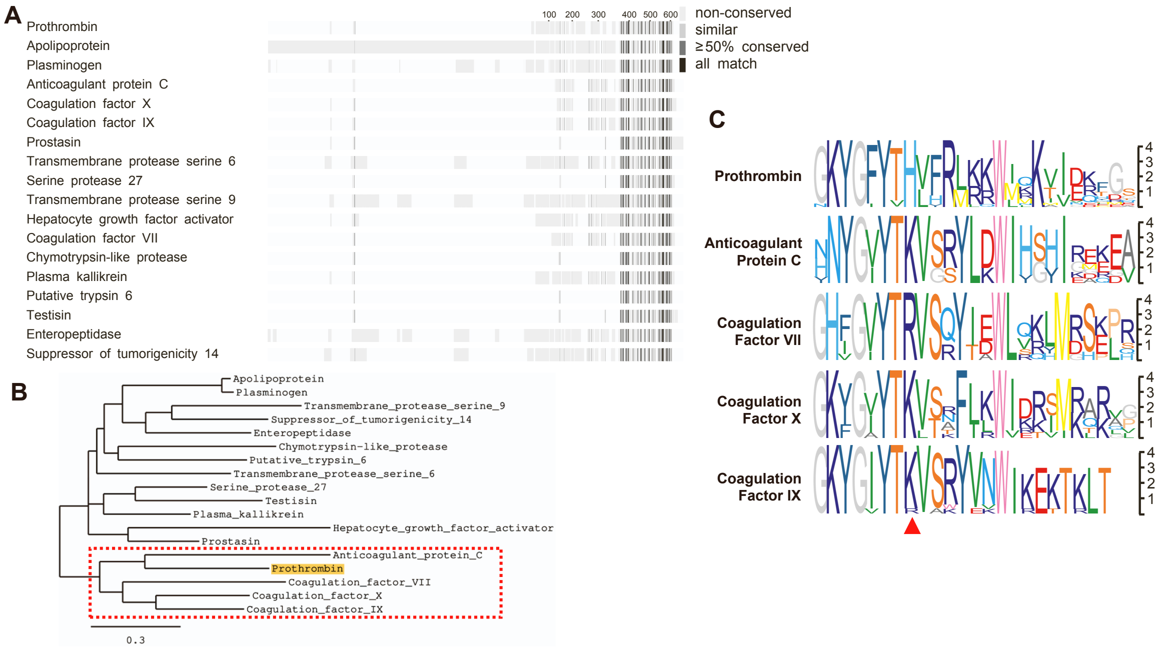

Figure S12 | Histidine is not conserved in thrombin homologs. (A) The sequence of human prothrombin (UniProt ID: P00734) was used to search for similar proteins in human in the UniProt database using the protein-protein BLAST algorithm (blastp). The figure shows sequence alignment of these proteins as fingerprints, whereby conserved regions are shown in dark grey. (B) Phylogenetic tree of prothrombin and related proteins based on the sequence alignment built using PhyML and TreeDyn algorithms (www.phylogeny.fr). Proteins that are in the same tree branch as prothrombin are highlighted in the dotted red box. (C) Sequences from different organisms for each protein in the same tree branch as prothrombin were searched on the UniProt database using the blastp algorithm and aligned to the sequences of prothrombin. The figure shows consensus sequence in WebLogo representation for residues that aligned with TCP25. The position of N-terminal histidine residue in HVF18 peptide is indicated by the red triangle.

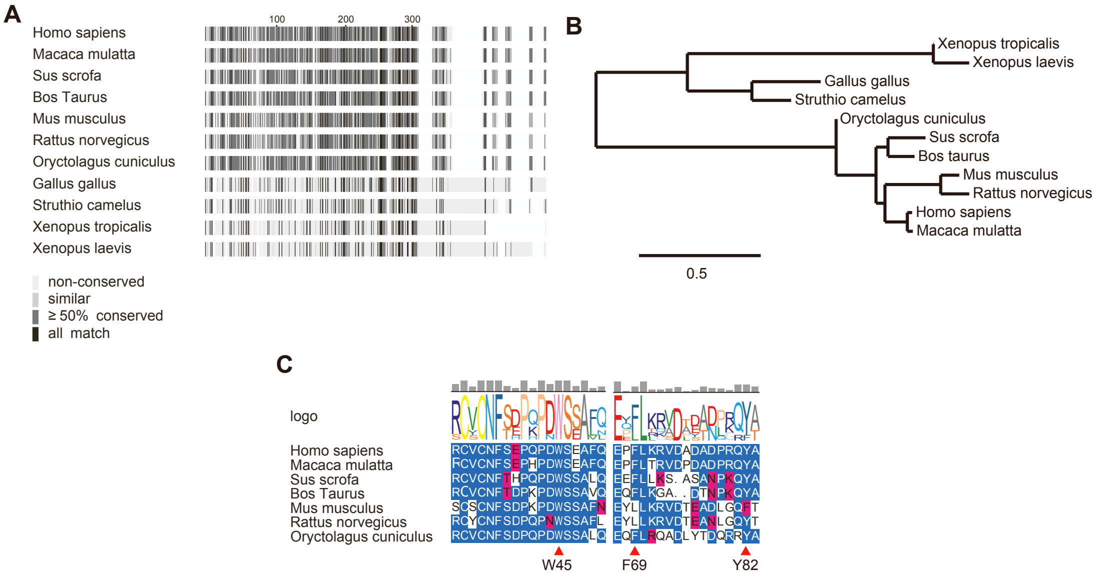

Figure S13 | Sequence conservation of CD14 in vertebrates. (A) Multiple sequence alignment of CD14 from various species represented as fingerprints with conserved sequence coloured in dark grey. (B) Phylogenetic tree of CD14 based on the sequence alignment built using PhyML and TreeDyn algorithms (www.phylogeny.fr). (C) Sequence alignment of N-terminal residues of CD14 coloured in blue for identical residues and magenta for similar residues. The degree of conservation is illustrated by the grey bars on top and the consensus sequence is shown in the middle as a WebLogo representation. The positions of residues that form cation-pi and staggered pi stacking interaction with peptide N-terminal arginine and histidine, respectively, are indicated by red triangles.

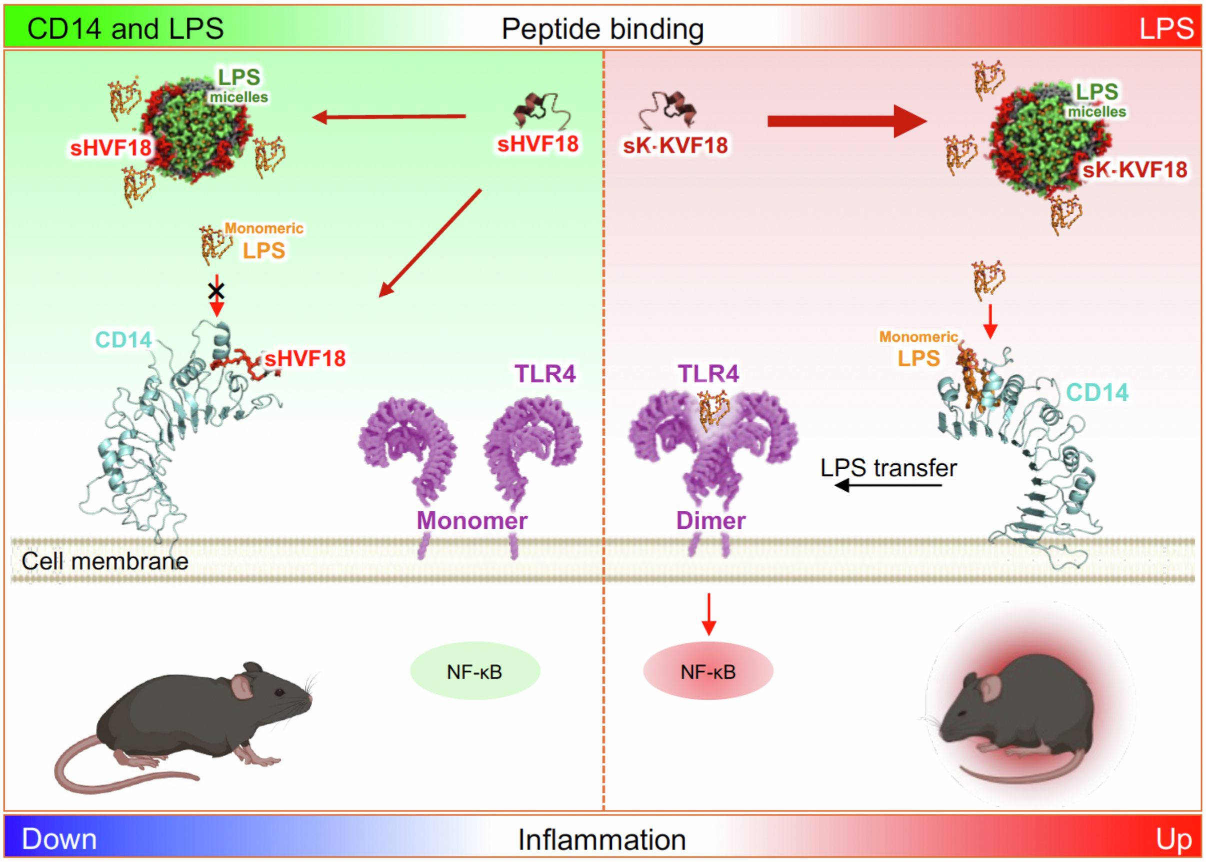

Figure S14 | Schematic figure illustrating how affinity of the peptide to LPS or CD14 and LPS affects inflammation in mice in CLP-induced sepsis. sHVF18, in addition to binding to LPS, also competes with LPS for the same site on CD14. This competition interferes with the CD14–LPS interaction, thereby hindering LPS transfer to TLR4, subsequent TLR4 dimerization, and downstream activation of inflammation through NF-κB. On the other hand, sK-KVF18, while binding strongly to LPS, does not bind to CD14 with the same affinity as LPS. As a result, free LPS after binding to CD14 is transferred to TLR4, enabling TLR4 dimerization and initiating inflammation through NF-κB.

A

Species	Accession number	F2		
		dN	dS	dN/dS
Macaca mulatta	NM_001104585.1	0.0219	0.1257	0.1742
Sus scrofa	NM_001122985.1	0.0846	0.4487	0.1885
Bos Taurus	NM_173877.1	0.0953	0.8445	0.1128
Mus musculus	NM_010168.4	0.1052	0.7744	0.1358
Rattus norvegicus	NM_022924.2	0.1159	0.8550	0.1356
Gallus gallus	NM_204605.2	0.2878	1.3932	0.2066
Struthio camelus	XM_009682749.1	0.2907	1.4866	0.1955
Xenopus tropicalis	NM_001015797.1	0.3247	2.5372	0.1280

B

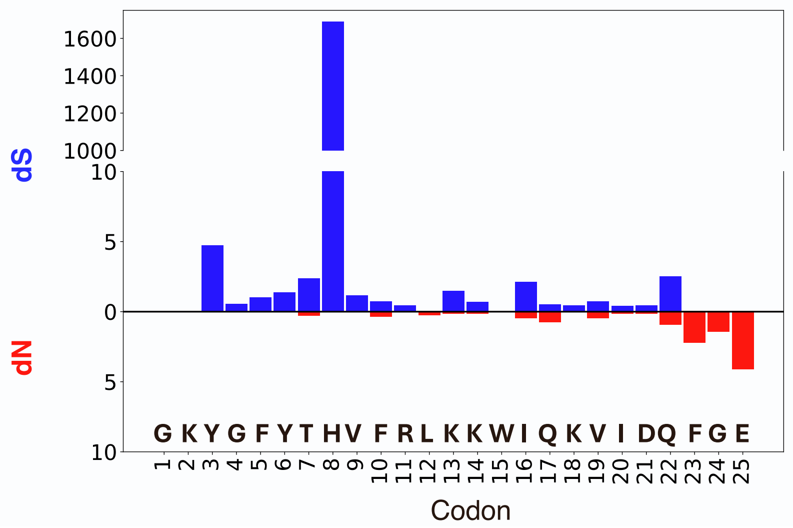

Figure S15 | Selection pressure on the F2 gene coding for thrombin. (A) Pairwise comparison of the full-length human F2 mRNA transcript sequence (GeneBank accession number: NM_000506.5) with other vertebrate sequences. The table shows the rate of non-synonymous substitution (dN), the rate of synonymous substitution (dS), and the ratio of dN/dS. Analysis was performed using the YN00 module in the PAMLX software. (B) Per codon maximum likelihood estimates of dS (blue) and dN (red) for the F2 transcript coding for the GKY25 peptide. Estimates above 10 are not shown. Resides coded by each codon in the human sequence are indicated. Analysis was performed using the Fixed Effects Likelihood (FEL) tool on Datamonkey webserver.
